# The discovered chimeric protein plays the cohesive role to maintain scallop byssal root structural integrity

**DOI:** 10.1038/s41598-018-35265-y

**Published:** 2018-11-20

**Authors:** Pingping Xu, Xiaoting Dai, Dandan Wang, Yan Miao, Xiaokang Zhang, Shuoshuo Wang, Luyao Teng, Bo Dong, Zhenmin Bao, Shi Wang, Qianqian Lyu, Weizhi Liu

**Affiliations:** 1 MOE Key Laboratory of Marine Genetics and Breeding, College of Marine Life Sciences, Ocean University of China, Qingdao, China; 2 Laboratory for Marine Biology and Biotechnology, Qingdao National Laboratory for Marine Science and Technology, Qingdao, China; 3 Laboratory for Marine Fisheries Science and Food Production Processes, Qingdao National Laboratory for Marine Science and Technology, Qingdao, China

## Abstract

Adhesion is essential for many marine sessile organisms. Unraveling the compositions and assembly of marine bioadheisves is the fundamental to understand their physiological roles. Despite the remarkable diversity of animal bioadhesion, our understanding of this biological process remains limited to only a few animal lineages, leaving the majority of lineages remain enigmatic. Our previous study demonstrated that scallop byssus had distinct protein composition and unusual assembly mechanism apart from mussels. Here a novel protein (Sbp9) was discovered from the key part of the byssus (byssal root), which contains two Calcium Binding Domain (CBD) and 49 tandem Epidermal Growth Factor-Like (EGFL) domain repeats. Modular architecture of Sbp9 represents a novel chimeric gene family resulting from a gene fusion event through the acquisition of CBD2 domain by *tenascin like* (*TNL*) gene from *Na*^+^*/Ca*^*2*+^ *exchanger 1* (*NCX1*) gene. Finally, free thiols are present in Sbp9 and the results of a rescue assay indicated that Sbp9 likely plays the cohesive role for byssal root integrity. This study not only aids our understanding of byssus assembly but will also inspire biomimetic material design.

## Introduction

Underwater bioadhesion is an essential biological process for marine sessile organisms, which is believed to be critical for movement, food procurement, self-defense, metamorphosis, and attachment^1–3^. Therefore, elucidation of marine bio-adhesive compositions provides the basis for understanding marine organism adhesion and its physiological roles. It is found that marine invertebrates have evolved highly diverse adhesion compositions to adapt their respective living environment. For example, adult barnacle cement proteins are released from single cells and then curing in seawater^4^. In contrast, mussel byssus relies on DOPA-rich proteins which self-assemble into complex architectures^5,6^. This apparent diversity presents the challenge in understanding of marine bioadhesion, therefore requiring to discover and characterize the key marine bioadhesives components in representative lineages.

In addition, exploration of the molecular evolutionary process of these proteins with unique mechanical properties will allow us to better understand their evolutionary origins. For example, phylogenetic analyses of the suckering teeth protein demonstrate that these genes originate from a common ancestor and are able to assemble into robust bulk biomaterials^7^. Therefore, obtaining the full-length sequence of key bioadhesive proteins is critical. However, no such protein has ever been identified directly from an marine animal’s genome through homology prediction^8^ indicating the potentially independent origin and evolution of many adhesive proteins in some (if not all) each animal lineages. How diverse adhesive proteins independently originated and evolved in aquatic animals remains a fascinating, unsolved question in the field.

It is known that some scallop species are able to attach to the substratum through a byssus with remarkably unique morphology^9^, although the scallop byssus is analogous to mussel byssus. However, the understanding of scallop byssus is still limited. Our previous study^10–12^ demonstrated that the protein composition of scallop byssus is intriguing as well. Therefore, it is very interesting and important to dissect the key scallop byssal proteins. Here by focusing on the byssal root, which has unique ultrastructure and the increased elasticity, a novel protein (Sbp9) was discovered and characterized. This Sbp9 was determined to be one of the most important components of scallop byssal root, and possesses a previously undiscovered modular organization. The EGFL/CBD (EC) protein represents an evolutionary innovation in the scallop lineage that has resulted in the development of novel functions. Taken together, these findings will aid the elucidation of the underlying byssus assembly and provide biological principles used for high-performance biomaterials design.

## Results and Discussion

### Root region of scallop byssus exhibits unique mechanical and ultrastructural properties

**Figure 1:**
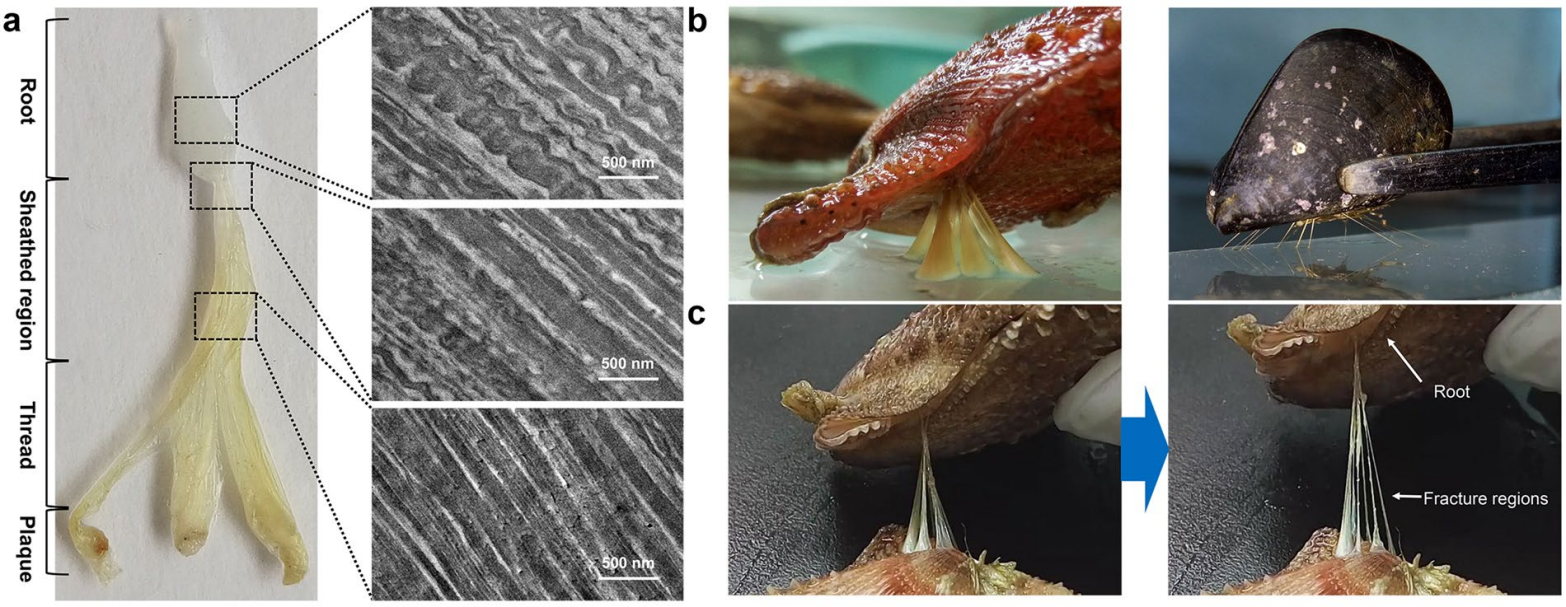
Scallop byssal root protein has unique structure and mechanical properties. (**a**) TEM photographs revealed that the composition of the root is different to that of the thread region, and this difference is correlated well with the gradual change in mechanical properties. (**b**) Differences of byssi between scallop and mussel. Scallop byssus is roughly concentric in the sheathed region, and the threads are separated from the end of the sheathed region, while mussel threads are separated from the stem. The amount of byssal protein secreted by scallop is greater than that secreted by mussel. (**c**) Mechanical measurements demonstrate that the root of scallop byssus can bear higher tensile strength than that borne by other regions.

Scallop byssal threads can be divided into four portions based on appearance and the ultrastructure (Fig. 1a). Both mechanical and ultrastructure analyses demonstrate a gradual transition along the byssal thread. Based on TEM, the scallop byssal root showed an obvious ultrastructural transformation along the thread. The proximal root exhibits coiled fibrils with a wavy structure, while the sheathed region displays a more densely packed fibrillar structure (Fig. 1a). This indicates that the scallop byssus may also display a gradual transition in its mechanical properties. This is similar to that in mussel^13^, although its morphology is significant different to the mussel byssus(Fig. 1b).

A detachment force assay showed that the scallop byssus is able to bear 119.58 ± 19.93 times of its body weight. Furthermore, although this root directly interacts with the scallop foot, fractures do not occur in the byssal root, while it happens at the interface between the thread and the plaque (Fig. 1c). In summary, these observations suggest that this root region with increased elasticity and toughness deserves to be explored and that the results will provide an in-depth understanding of scallop byssus assembly and its function; however, studies of this region are rare.

### Identification of the key proteins in scallop byssal root

**Figure 2:**
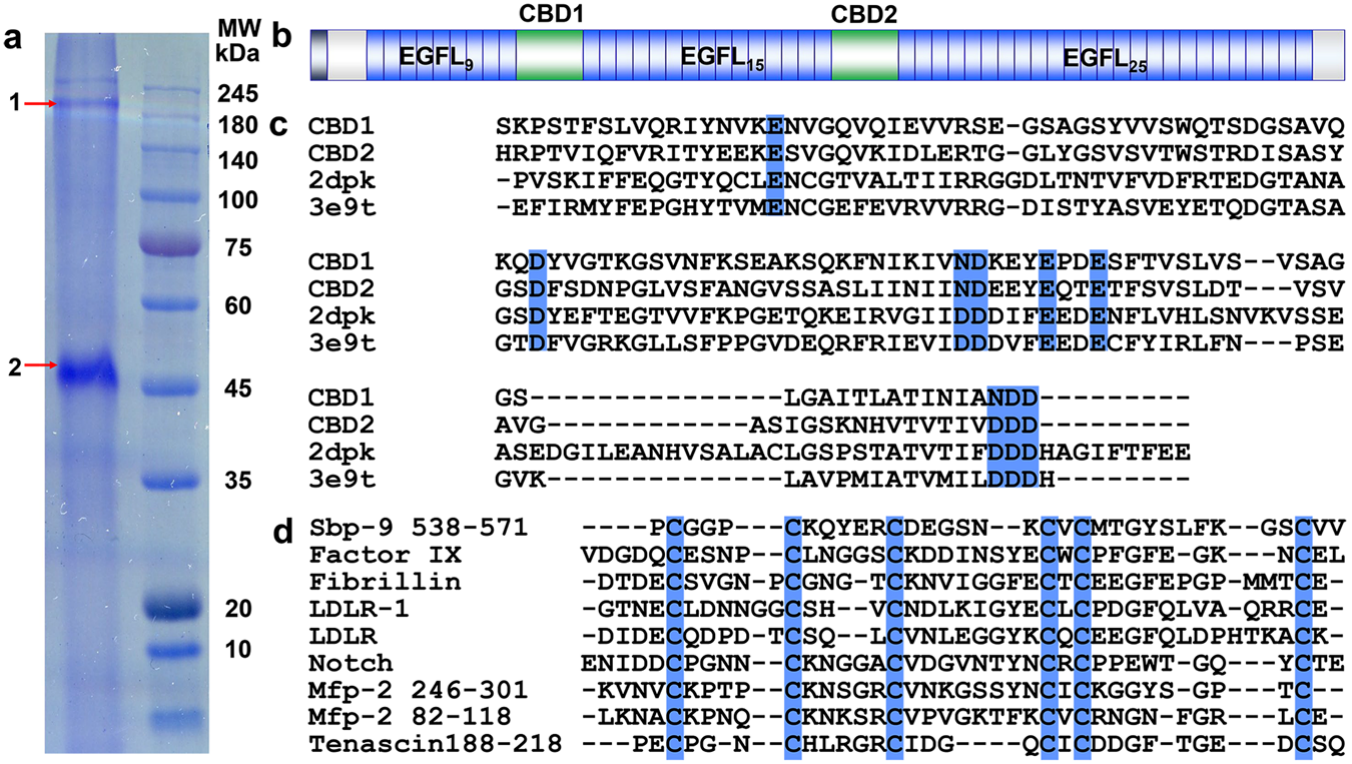
Discovery of a novel scallop byssal root protein. (**a**) SDS-PAGE of extract from the scallop byssal root showing two major fractions of high and medium molecular weights when stained with Coomassie Brilliant Blue R-250. To explore the compositions of the root, the corresponding fractions were analyzed by mass spectroscopy. (**b**) Schematic view of the main structural domains of Sbp9. The domain annotation predicted by SMART. EGFL, EGF-like domain; CBD, calcium-binding domain. The conserved and unique PCGGPC domain is highlighted. (**c**) Multiple sequence alignment of CBD1^Sbp9^ against other CBD domains for which the structures have been determined. These domains include the CBD1 domain (PDB:2dpk) from *C. lupus* NCX1 and the CBD1 domain (PDB:3e9t) from *D. melanogaster* calx with sequence identity >30%. The alignment shows that CBD1^Sbp9^ contains conserved Ca^2+^-binding residues, as highlighted in blue (E^405^, D^440^, N^465^, D^466^, E^470^, E^473^, N^500^, D^501^, D^502^). (**d**) Sequence alignments of various EGFLs including Factor IX, Fibrillin-1, LDLR, Notch, Mfp-2 and Tenascin-X. Other EGFL-rich proteins from mussel adhesive proteins (Mfp-2) were also used in the alignment. Conserved Cys residues are highlighted by blue.

To reveal the protein composition of the root, protein was extracted from the scallop byssal root (Fig. 1a). Based on the SDS-PAGE, two major bands were observed (Fig. 2a). These two bands (band 1 and 2) were cut for in-gel digestion and subjected to mass spectroscopy analysis respectively. The mass spectrometry raw data were searched against the translated *C. farreri* full protein databases (BioProject accession: PRJNA185465)^11^. In total, 21 proteins with unique protein peptides ≥2 were identified from scallop byssal root extractions (Supplementary Tables S1 and S2). Furthermore, two other criteria were considered to define the potential key proteins: (i) The proteins should be foot-specific (seven of the proteins were foot-specific); (ii) The protein should be an abundant constituent in the root (semi-qualitative MS analysis demonstrated that a protein with contigs (52787.5, GenBank accession number: MK050192) is the most abundant proteins in the top band (band 1) and is the third most abundant protein in the lower band (band 2) (Fig. 2a and Supplementary Tables S1 and S2). Accordingly, the protein encoded by 52787.5 was identified as the key component of the scallop byssal root and was named Sbp9 in the following study. The detection of Sbp9 in lower band (band 2) suggest protein was degraded.

However, we failed to obtain the full length sequence based on the determined *C. farreri* genome^11^, possibly due to the presence of the tandemly repeated motif. To solve this issue, the full-length Sbp9 gene sequence was further explored using on the Pacbio Sequel sequencing instrument as described in Supplementary Data1. It turned out that the full length *Sbp9* contains 6099 bp, encoding 2033 amino acids. And the annotation and functional domain analyses based on Simple Modular Architecture Research Tool (SMART, http://smart.embl-heidelberg.de/) show that Sbp9 contains two CBD domains (CBD1 and CBD2) with a flanking 49-EGFL repeats domain (Fig. 2b). CBD domains found in Na^+^/Ca^2+^ exchangers can bind Ca^2+^ and act as Ca^2+^ sensors^14^. In addition, the CBD domain was found to be involved in a protein–protein interaction platform during cell adhesion and signaling events^15^. Sequence alignment was performed for the two CBD in Sbp9 with several CBD domain structures from different species (PDB:2dpk, the first CBD of *Canis lupus* NCX1^16^; and PDB: 3e9t, the first CBD of the *Drosophila melanogaster* Na^+^/Ca^2+^ exchanger, Calx^17^) (Fig. 2c), which suggests that CBD1^Sbp9^ and CBD2^Sbp9^ contains conserved Ca^2+^-binding residues. On the other hand, sequence alignment among EGFL showed the low sequence similarity between the EGFL in Sbp9 and other EGFLs (Sequence identity <30%) (Fig. 2d). To study the sequence features of the EGFL derived from Sbp9, multiple sequence alignments among all 49 EGFL domains were carried out, and the results show that one significant feature of these conserved EGFL repeats is the presence of the PCGGPC motif at the first two Cys residues (Supplementary Fig. S3), which is quite similar to the predominant subrepeat of GPGGX in flagelliform silk^18^. Further sequence alignment against other well-characterized EGFL domains (Fig. 2d) also demonstrates that the PCGGPC motif is a significant feature of EGFLs derived from Sbp9. Multiple EGFL domain-containing proteins have previously been discovered in mussel byssal protein (Mfp-2) and are believed to play an important “cohesive” role for mussel byssus assembly^19^. However, the EGFL/CBD fusion domain architecture has not been found in animals outside the scallop lineage (see discussion below).

**Figure 3:**
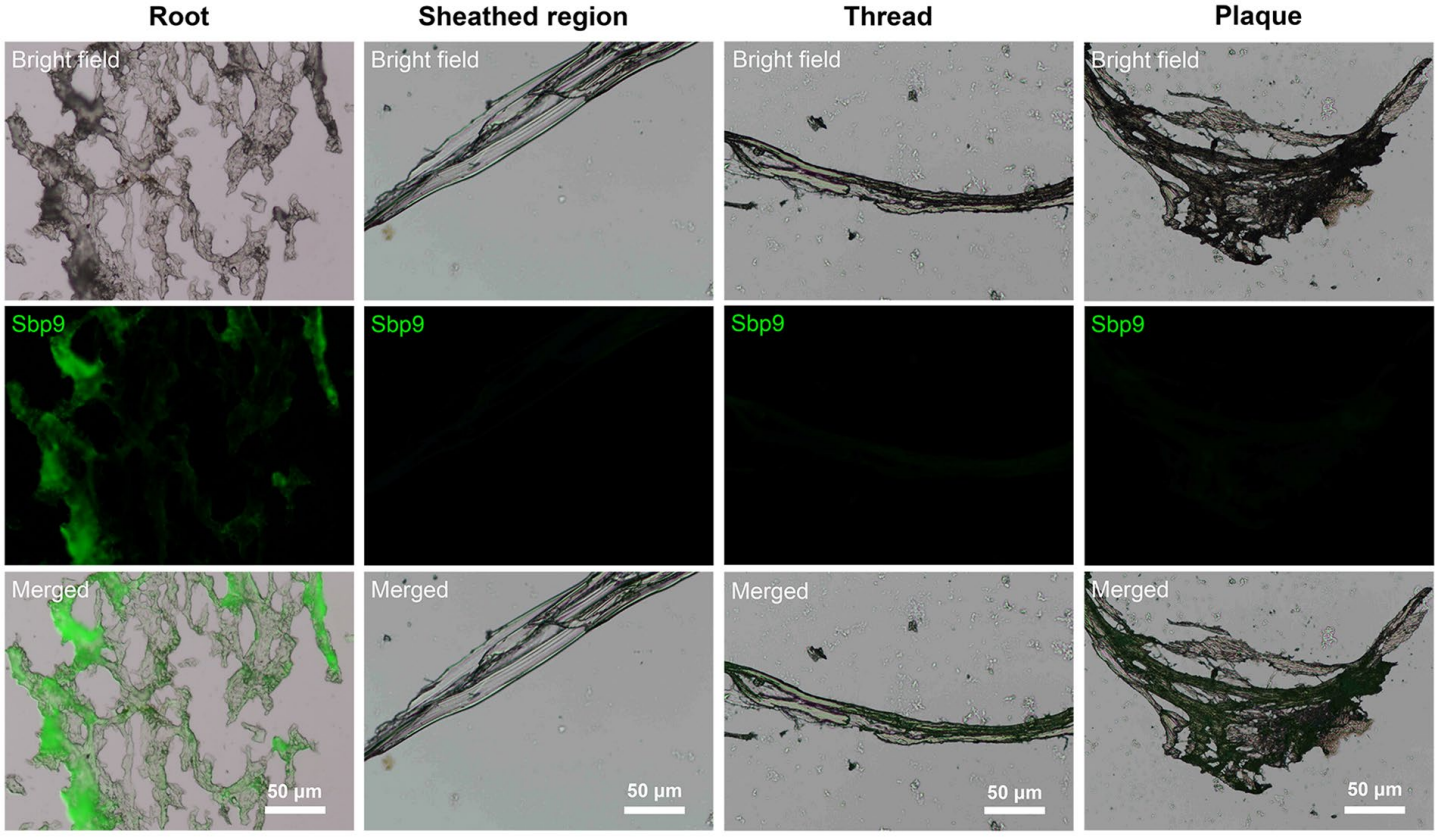
Immunohistochemical analysis demonstrates that Sbp9 is mainly located in the root.

To further investigate the distribution of Sbp9 in byssus, immunohistochemistry was carried out using polyclonal anti-CBD1 antibodies. The results showed that the fluorescence signal was mainly restricted to the root, and little signal was found in the other regions (Fig. 3). This observation suggests that Sbp9 is mainly located in the byssal root. In summary, the above comprehensive analyses indicate the presence of uncharacterized Sbp9 protein with the unique domain architecture, however the role of Sbp9 in scallop byssal root remains enigmatic.

### Genome-wide characterization and evolutionary analysis of EGFL/CBD (EC) genes

**Figure 4:**
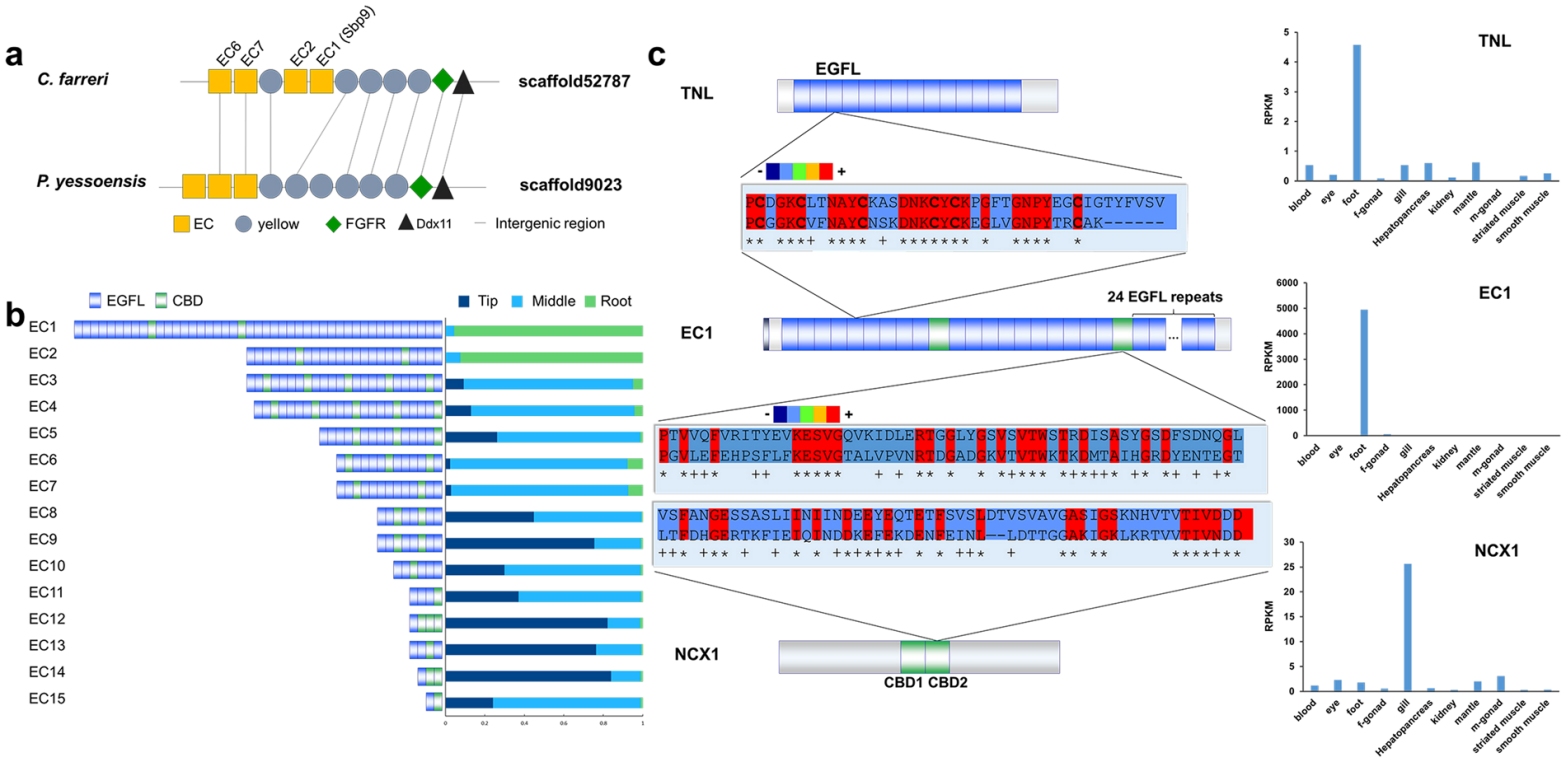
Characterization and evolutionary analysis of *EC* genes. (**a**) Genomic distribution of *Sbp9* and related *EC* genes. Genes with homologous annotations are displayed. (**b**) Gene structures and across-foot expression profiles of all *EC* genes in the *C. farreri* genome. The horizontal axis of the histogram represents the relative expression levels of three foot subregions. (**c**) Possible evolutionary origin of the scallop *EC* gene. Protein sequences of related domains were aligned using the Megalign program^32^. Different colors indicate the degree of matching between each aligned sequence: blue, 21–40%; red, 81–100%.

To understand the evolution of scallop *EC* genes, we conducted a genome-wide search using Sbp9 as a query sequence and identified 15 *EC* genes in the *C. farreri* genome (Supplementary Data2). Genomic distribution analysis revealed that these genes were often physically in close proximity (Fig. 4a and Supplementary Fig. S2), suggesting that some of them (including *Sbp9*) may originate from gene duplication. Comparison with another scallop species, *P. yessoensis*, revealed that *Sbp9* and *EC2* are *C. farreri*-specific and may have functionally diverged from *EC6* and *EC7* due to their distinct across-foot expression profiles (see Fig. 4b). Among the 15 *EC* genes, the EGFL/CBD domain numbers are highly variable, ranging from 1/1 to 43/5. There is a tendency for *EC* genes possessing more EGFL/CBD domains to be preferentially expressed in the middle and root of scallop foot, while those with fewer domains are preferentially expressed in the tip region (Fig. 4b). In particular, *Sbp9* shows predominant expression in the root, similar to that of *EC2* but quite different from those of other *EC* genes.

The unique EGFL/CBD domain structure of *EC* genes is not found in animals outside the scallop lineage, suggesting that this gene family represents an evolutionarily innovation in the scallop lineage. To understand the evolutionary origin of EGFL/CBD domains, domain similarity searching against the whole genome was conducted, which revealed that the EGFL and CBD domains of the *EC* gene exhibit the highest protein homology to the EGFL domain of the *tenascin-like* (*TNL*) gene and the CBD2 domain of *Na*^*+*^*/Ca*^*2+*^ *exchanger 1* (*NCX1*) gene, respectively (Supplementary Table S3). Interestingly, the *TNL* gene is both structurally and expressionally similar to the *EC* gene (i.e., regarding the tandemly arrayed EGFL domains and foot-specific expression). This might suggest that the *EC* gene represents a chimeric gene, the emergence of which might have occurred through the acquisition of the CBD2 domain from *NCX1* and EGFL repeats from *TNL* (Fig. 4c).

Our study suggests that *EC* genes represent an evolutionarily innovation in the scallop lineage, the emergence of which is likely due to a gene fusion event that occurred through the acquisition of the CBD2 domain by *TNL* from *NCX1* (Fig. 4c). Gene fusion represents one of several important ways for the emergence of new genes^20,21^, and a novel combination of domains can result in a range of post-translational outcomes, including domain relocalization, new inter-protein associations, the regulation of enzymatic activity and possibly even the formation of novel protein functions^22,23^. The potential evolutionary advantage of obtaining CBD domain by scallop *EC* genes is to increase the sensitivity of tandemly EFGL domain to the presence of calcium, which was identified as one of two metals that trigger the regulation of mussel byssal cohesiveness through EGFL repeat-containing Mfp-2^19^. Interestingly, it has been shown that vertebrate *TN-X* genes can regulate the spacing and cohesiveness between collagen fibrils through direct or indirect interactions^24^. This could suggest an ancestral role of *TNL* in the regulation of fibrous cohesiveness and possibly explains why this gene was co-opted for the regulation of byssogenesis in the scallop lineage. In addition, this finding might be promising for the rational molecular design of biomimetic materials, which is yet challenging but important for biomaterials development. Previously a multi-component nanofibers were designed to acquire a strong underwater adhesive by fusing the Mfp5 with the CsgA^25^.

### Rescue assay suggests the cohesive role of EGFLs in byssal root

**Figure 5:**
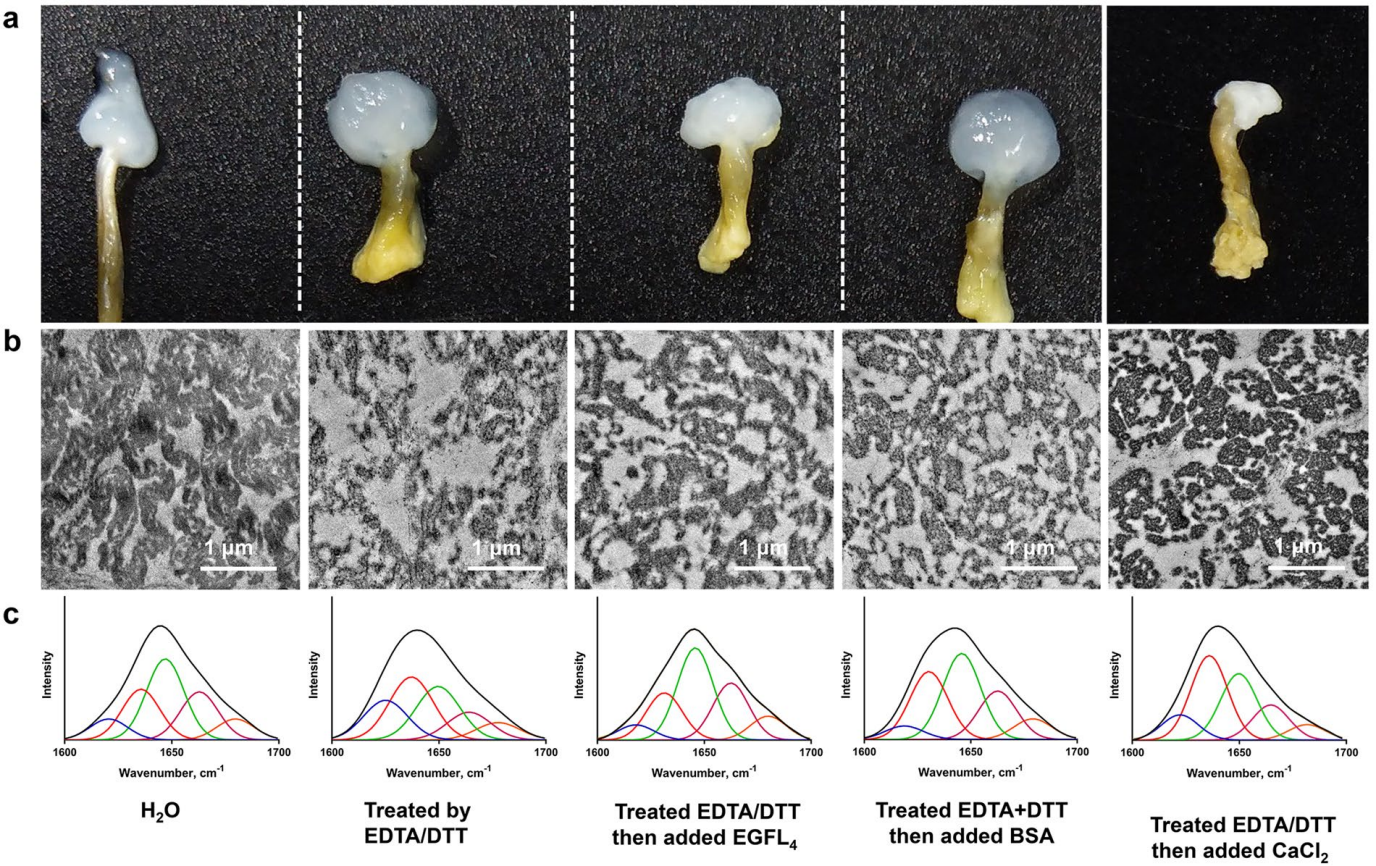
Rescue assay showing that Sbp9 is indispensable for the integrity of scallop root. (**a**) EGFL_4_ partially recovered byssal root integrity. The native byssal root was whitish, and the root became more transparent with EDTA/DTT treatment when compared to the untreated group. The addition of EGFL_4_ or Ca^2+^ caused the root to become whitish, while the addition of BSA did not. (**b**) TEM photographs revealed that the addition of EGFL_4_ partially repaired the byssal root. The native byssal root comprises uniform fibers, as shown by the untreated group. EDTA/DTT treatment resulted in the byssal root proteins agglutination together irregularly, and the fibers became loose. The addition of EGFL_4_ partially rescued the byssal root integrity, while the addition of BSA did not. The addition of Ca^2+^ partially rescued the fibers cohesive while high electron-dense granules were still presence. (**c**) FTIR spectra revealed that addition of EGFL_4_ is able to partially restore the architecture of the disrupted byssal root by the EDTA/DDT treatment. EDTA/DTT treatment altered the secondary structure of byssal root proteins. The addition of EGFL_4_ partially rescued the secondary structure, while the addition of BSA or Ca^2+^ did not.

In canonical EGF-like domains, six conserved Cys residues form three disulfide bonds^26^. However, our Ellman’s assay results showed the presence of free thiol in EGFL_4_, which is different to the case in canonical EGF-like domains (Supplementary Table S4). Therefore, to examine the roles of Sbp9 containing the EGFL_4_ module in byssus, the EGFL_4_-restoring experiment was conducted. EDTA/DTT treatment was applied to destroy the possible interactions within the scallop byssal root. The byssal root became more transparent with the EDTA/DTT treatment, suggesting that the ultrastructure of the root was destroyed. Further TEM visualization demonstrates that the uniform fibers became loose, which resulted in the architecture of byssal root fibers destroyed (Fig. 5b). Also, the ratio of coils in byssal root proteins was decreased and the ratio of β-sheets was increased (Fig. 5c and Supplementary Table S5) indicates that the EDTA/DTT treatment disrupted the integrity of byssal root, and intermolecular interactions within the fibers in the root were disrupted.

Previous study demonstrated that Ca^2+^ is able to stabilize nanocavities in *Pinctada fucata* byssus^27^. In order to compare effect of Ca^2+^ in the disassembly of byssal proteins, the EGFL_4_ or Ca^2+^ was added to the EDTA/DTT-treated samples respectively. Apparently the addition of Ca^2+^ was able to partially restore the architecture between byssal root fibers, while irregular high electron-dense granules was still presence, and no significant secondary structure change was observed compared with EDTA/DTT treated group (Fig. 5 and Supplementary Table S5). And addition of EGFL_4_ was able to partially recover the natural ultrastructure, and secondary structure almost recovered the natural state (Fig. 5 and Supplementary Table S5). Therefore, it is concluded that when EGFL_4_ or Ca^2+^ added, the byssal root partially recover the natural structure (Fig. 5 and Supplementary Table S5). However, no significant change was observed for the BSA control (Fig. 5 and Supplementary Table S5). It is therefore reasonable to conclude that EGFL domain derived from Sbp9 is able to partially rescue the disrupted root structure. One possible explanation is the presence of extra EGFL_4_ sacrifices itself by consuming residual DTT, therefore, the byssal root region is able to partially recover the original structure. Also, another possible explanation is free Cys in the EFGL_4_ can form covalent bonds with neighboring byssal fibers through formation of either the disulfide or the Dopa-quinone^28^, which both extensively exist in scallop byssus^11^. Extensive studies have indicated that TN-X, an EFGL-containing protein in ECM, primarily plays an architectural role and is able to interact with fibrillar collagens^24^. Furthermore, previous study has shown that the EGFL domain in TN-X is important for the interaction with collagen^29^. These observations suggest that Sbp9 may play a cohesive role as the cross-linker that is responsible for structural integrity of scallop byssal root.

### A model was proposed to describe the role of Sbp9 in scallop byssal root

**Figure 6:**
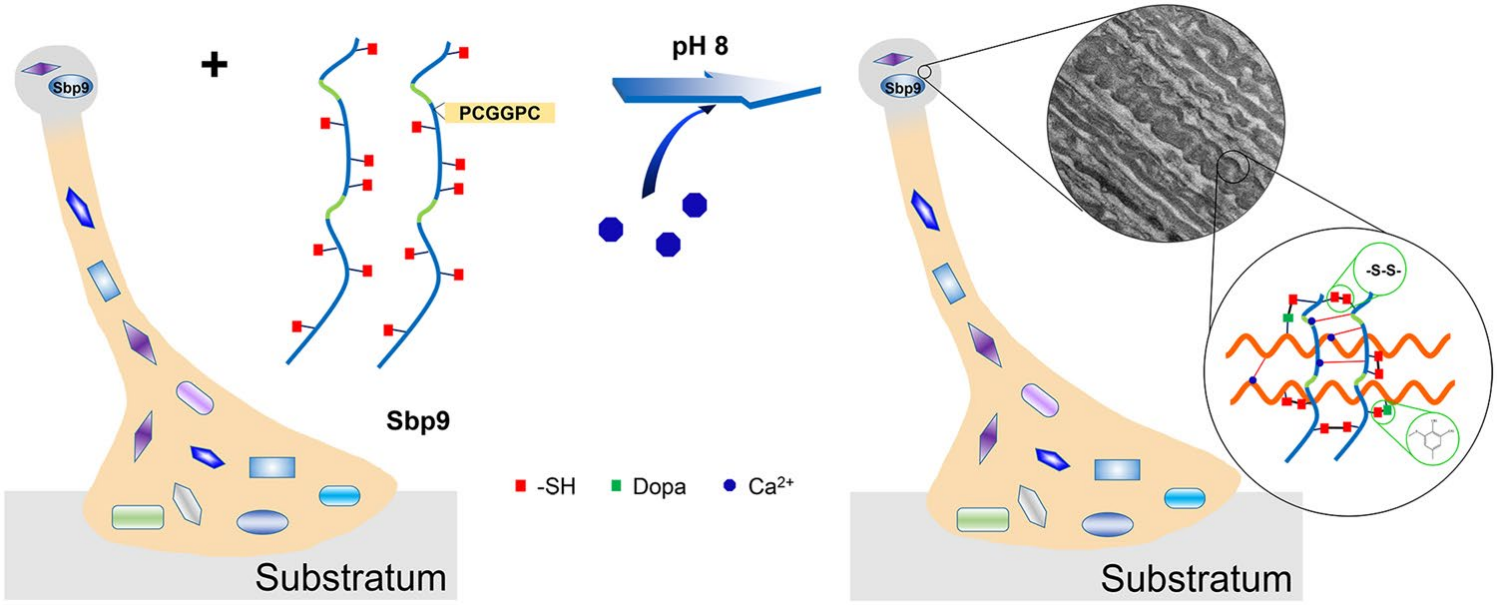
Possible cohesive role of Sbp9 in byssal root. Sbp9 is able to cross-link with other byssal root proteins through the formation of intermolecular disulfide bonds with free thiol (red box) and the formation of cysteinyldopa with DOPA (green box) in the seawater environment (in the presence of Ca^2+^ at pH 8). Ca^2+^ (blue octagons) is able to alter the protein self-assembly properties to further strengthen these interactions.

Taken together, it was found that Sbp9 has evolved to possess a unique modular architecture, the EGFL/CBD fusion protein (Fig. 1e). The CBD domain is a well-known Ca^2+^-binding protein and it most likely acts as a calcium sensor^14^. And the multiple tandem EGFLs are able to self-assemble into packed fibrils through the free thiol, which then covalently interacts with other fiber components in the root (Fig. 6). Based on the above analyses, a model for the role of Sbp9 in the scallop byssal root region is described in Fig. 6. During the formation of byssus, Sbp9 is able to cross-link with other components in the byssal root, especially byssal root proteins, which possibly lead to elasticity increase. Extensive interactions may occur, including the disulfide bond between the free thiol and the cysteinyldopa between the free thiol with Dopa, as detected in the scallop byssus^10,11^. Simultaneously, metal ions present in seawater, especially Ca^2+^, are able to alter the protein self-assembly properties to further strengthen these interactions and can function as a “trigger”.

## Conclusions

Focusing on the important but unexplored scallop byssal root, a key protein (Sbp9) with a unique modular architecture was identified, the emergence of which is possibly related to a gene fusion event. Free thiols are present in Sbp9 and the results of a rescue assay indicate that Sbp9 plays a cohesive role to maintain the integrity of the scallop byssus. For the organisms themselves, they have evolved to create these robust materials to adapt the living environments. In summary, this study provides the first comprehensive characterization of a key scallop byssal protein and will facilitate understanding the assembly of scallop byssus; in addition, the characterized unique module architecture can potentially be used in the design of biomimetic materials.

### Experimental procedures

#### Ultrastructure observation of scallop byssus

Scallops were purchased from a seafood market in Qingdao and were cultured under laboratory conditions (~19 °C) for 24 hrs before the foot was harvested. Byssus secreted over 24 hrs were harvested and fixed in 2.5% glutaraldehyde overnight followed by 1% osmic acid for 1 h. The samples were dehydrated in serial acetone solutions (30, 50, 70, 80, 90, 95%) and finally in 100% acetone. The dehydrated samples were then infiltrated with 3:7 and 7:3 acetone: Spurr and then cured in Spurr resin at 60 °C for 24 hrs. Subsequently, different byssal regions were sectioned using a microtome to produce 70-nm-thin transverse or longitudinal sections. All the transverse sections were taken from in the center of the byssal root. Finally, the sections were stained with 2% uranyl acetate for 10 min and examined under a Hitachi H-7650 TEM (Hitachi, Tokyo, Japan) operated at 100 kV. For the EDTA/DTT treatment assay, byssi were treated in 0.2 M EDTA/50 mM DTT for 12 hrs, and EGFL_4_ restoring experiments were performed by immersing EDTA/DTT-treated byssus in 20 mM CaCl_2_, 1 mg/mL EGFL_4_ or BSA. Total number of irregular high electron-dense granules were counted in 10 randomly selected 200 × 200 nm^2^ square. The data are expressed as the mean ± S.D.

#### Identification of scallop byssal root protein compositions based on a proteomics approach

Byssi were harvested every 24 hrs as described above. The root was harvested as depicted in Fig. 1a, and this fraction was rapidly frozen in liquid nitrogen and ground under nitrogen using a small hand-held tissue grinder. The soluble fraction was extracted with 5% acetic acid (v/v) containing 6 M GdnHCl at 37 °C for 1 hr and freeze-dried after dialysis against deionized water at 4 °C overnight. The proteins were dissolved in 8 M urea and resolved in 10% (w/v) SDS-PAGE gels, which were then stained with Coomassie Blue R-250. The main protein gel bands were collected for mass spectrometry using an Orbitrap Elite mass spectrometer (Thermo Fisher Scientific, San Jose, CA, U.S.) as described previously. The raw files were analyzed using Proteome Discoverer 1.4 software (Thermo Fisher Scientific). To estimate protein abundance, MaxQuant (Version1.5.0.3) (Max Planck Institute, MPI, Martinsried, Germany) was used. A search for the fragmentation spectra was performed using the MASCOT search engine embedded in Proteome Discoverer against the *C. farreri* full-protein database^11^. The following search parameters were used: monoisotopic mass, trypsin as the cleavage enzyme, carbamidomethylation of cysteine specified as fixed modifications, and the oxidation of methionine, and the phosphorylation of tyrosine, serine or threonine was specified as a variable modification. The proteins were identified by Shanghai Omicsspace Biotechnology Co., Ltd. (http://omicsspace.com/).

#### Immunohistochemistry

Fresh byssus was collected within 2 hrs of deposition. After fixing with 4% paraformaldehyde (w/v) in PBS at room temperature for 2 hrs, byssi were dehydrated in 30% sucrose solution until they sank. Byssi were then embedded in opti-mum cutting temperature compound (SAKURA, Torrance, U.S.), and a parallel series of 10-µm sections were obtained using a freezing microtome (Leica CM 1850, Heidelberger, Germany). Antigen retrieval of the sections was performed by microwave heating in 10 mM sodium citrate buffer, pH 6.0. The sections were pre-incubated in blocking buffer (PBS containing 10% goat serum) for 30 min and then incubated at room temperature for 5 hrs with rabbit anti-CBD1. The primary antibody was labeled with Alexa Fluor® 488-conjugated goat anti-rabbit IgG (ZSGB-Bio, Beijing, China) at 37 °C for 1 hr and observed under a Nikon ECLIPSE NI fluorescence microscope (Nikon, Tokyo, Japan). Control sections were treated with the same protocol but omitting the primary antibody.

#### Genome-wide identification and evolutionary analysis of *EC* genes

To identify all potential scallop *EC* genes, we searched the Sbp9 protein sequence against the full protein databases of two scallop species (*Chlamys farreri*^11^ and *Patinopecten yessoensis*^30^) using an e-value threshold of 1e-05. The identified *EC* genes were further checked for the presence of EGFL and CBD domains. The EGFL repeats were determined using the Simple Modular Architecture Research Tool, SMART (http://smart.embl-heidelberg.de/) program and the spacing and number of their characteristic cysteine residues (XCX_3_CX_5-6_CX_4–6_CXCX_10–13_CX_2–8_). The CBD domain was determined by the NCBI Conserved Domain program (https://www.ncbi.nlm.nih.gov/Structure/cdd/wrpsb.cgi) and was confirmed using the SMART. Only candidate genes that contained both EGFL and CBD domains were qualified as *EC* genes. To investigate the potential evolutionary origins of scallop *EC* genes, the EGFL and CBD domain sequences of *EC* genes were searched (BlastP) against all other genes in the *C. farreri* genome, and the candidate *non-EC* genes with the highest alignment score and identity were selected for further investigation. The identified protein sequences were curated using RNA-seq data^11^ if a sequencing and/or assembly error existed.

#### Fourier transform infrared spectroscopy (FTIR)

The scallop byssi were treated by EDTA/DTT and EGFL_4_ or BSA-restoring as described in the method Ultrastructure observation of scallop byssus and then freeze-dried. The secondary structure of scallop byssal root proteins was measured by attenuated total reflectance Fourier transform infrared spectroscopy (ATR-FTIR) using a Nicolet™ iN™10 FTIR Microscope (Thermo Scientific) as previously described^27,31^. All spectra were recorded with a resolution of 2 cm^−1^, and the area under the amide I region (1700–1600 cm^−1^) were decomposed with PeakFit 4.12 software. Local minima in the second derivative were used to identify peaks, which were fit as Gaussian curves. The spectra were characterized by strong absorbance at approximately 1610–1640 cm^−1^ assigned to β-sheet structures, 1640–1648 cm^−1^ assigned to coils, 1648–1660 cm^−1^ assigned to α-helix, 1660–1685 cm^−1^ assigned to β-turn of the amide I region.

## Electronic supplementary material

Supplementary Information
