# Supplementary material for: The discovered chimeric protein plays the cohesive role to maintain scallop byssal root structural integrity
Source: Sci Rep. 2018 Nov 20;8:17082. doi: 10.1038/s41598-018-35265-y (PMC6244088; doi:10.1038/s41598-018-35265-y)
Supplement: Supplementary file 1 — Supplementary Information [file 41598_2018_35265_MOESM1_ESM.pdf]

The discovered chimeric protein plays the cohesive role to maintain scallop byssal root structural integrity

Pingping Xu[1,2,+], Xiaoting Dai[1,+], Dandan Wang[1], Yan Miao[1], Xiaokang Zhang[1], Shuoshuo Wang[1], Luyao Teng[1], Bo Dong[1,2], Zhenmin Bao[1,3], Shi Wang[1,2], Qianqian Lyu[1,2,*], and Weizhi Liu[1,2,*]

[1]MOE Key Laboratory of Marine Genetics and Breeding, College of Marine Life Sciences, Ocean University of China, Qingdao, China;

[2]Laboratory for Marine Biology and Biotechnology, Qingdao National Laboratory for Marine Science and Technology, Qingdao, China;

[3]Laboratory for Marine Fisheries Science and Food Production Processes, Qingdao National Laboratory for Marine Science and Technology, Qingdao, China.

***corresponding.author:**

Weizhi Liu. E-mail: liuweizhi@ouc.edu.cn

Qianqian Lyu. E-mail: lqqdo@163.com

[+] These authors contributed equally to this work.

Supplementary methods

Mechanical measurements for scallop byssus. To measure the mechanical strength of byssus, adult *C. farreri* were purchased from an aquatic farm in Qingdao; scallops adhering by scallop byssus were fixed to cages and dragged by a dynamometer. In total, 10 scallops were randomly selected for tension measurement to explore the fracture region.

Byssus detachment force was calculated as the max tension/scallop weight. Data are expressed as the means ± S.D.

Full length determination for *Sbp9*. cDNA of *Sbp9* was synthesized using the SMARTer^{TM} RACE cDNA Amplification Kit (Clontech, CA, USA) according to the protocol. The primer for 5'-RACE was TGGTAGCCTGGGTTACACTGACACTTGTT and the primer for 3'-RACE was TATGGTTGGCAATCCCTACTCCCG. The products were cloned into a pMD18T vector (Takara, Kyoto, Japan). The sequence of 5'-RACE product was confirmed through Sanger sequencing and the sequence of 3'-RACE product was confirmed through Sanger sequencing and third-generation sequencing. The sequencing libraries for the third-generation sequencing were prepared with the standard protocol from PacBio. Briefly, DNA was amplified by PCR. After fragment DNA, repairing DNA damage and DNA ends, anneal sequencing primer to SMRTbell^{TM} templates, target DNA was sequenced by Oebiotech (http://www.oebiotech.com/) on the Pacbio Sequel sequencing instrument to overcome the multiple repeats issues.

BLAST 2.3.1+ software was used to compare sequences from SMRT sequencing and sequences from Sanger sequencing. The consensus sequences from BLAST were analyzed by the software MEGA7 (http://www.megasoftware.net/) and the final sequence was acquired by correction based on the multiple sequences through insert the missing bases and/or delete the redundant bases.

Expression profiling of EC and related genes in scallop foot and other organs/tissues. The expression levels of EC and related genes were retrieved from the published RNA-seq datasets of *C. farreri* [1], including eleven adult organs/tissues (blood, eye, foot, female gonad, gill, hepatopancreas, kidney, mantle, male gonad, striated muscle, smooth muscle) and three foot subregions (tip, middle and root). Each organ or tissue was represented by three biological replicates. The expression value was calculated using the TMM algorithm implemented in EdgeR software[2] and was represented as reads per kilobase per million mapped reads (RPKM).The expression levels of EC and related genes

are represented by an average RPKM of three biological replicates.

Polyclonal antibody preparation. Polyclonal antibody was prepared and purified by ABclonal Biotechnology (Wuhan, China). Three rabbits were injected with $CBD1^{Sbp9}$ as described in the methods under **Recombinant protein over-expression and purification**; the protein was dissolved in PBS and complete Freund's adjuvant in a ratio of 1:1, and the rabbits were boosted three times (at weeks 3, 6, and 9). Affinity chromatography was utilized for antibody purification.

Evaluation of the chemical form of the Cys residues. The amount of nonoxidized Cys residues was quantified spectrophotometrically using Ellman's reagent (5,5'-dithiobis (2-nitrobenzoic acid), DTNB), which reacts with thiol groups of free thiol to yield2-nitro-5-mercapto-benzoic acid (TNB)[3]. $EGFL_2$and $EGFL_4$were stood overnight at ambient temperature (~ 25 °C). Then, 50 μL of 10 mM DTNB were added to 2.45 mL of 20 mM Tris-HCl pH 8.5 buffer containing ~1 mg/mL protein. The amount of thiol remaining in the reaction medium was quantified by measuring TNB absorbance at 412 nm. L-cysteine was performed as a standard and lysozyme, of which the eight Cys residues form four disulfide bonds, was performed as a negative control.

Recombinant protein over-expression and purification. To enhance the yield of the recombinant proteins, the codon optimization was carried out and the corresponding genes were synthesized. The CBD1 and $EGFL_4$ fragment were obtained by PCR amplification using the synthesized $CBD1^{Sbp9}$-$EGFL_4$ as template. All the primers were provided in Table S6. Then the fragments were digested with restriction enzymes (*BamH*I and *Xho*I), before they were inserted into the modified pET-32-HisTT (modified from Novagen pET-32) vector. All the recombinant constructs were verified by DNA sequence.

To over-express the recombinant interest protein, these constructs were transformed into *E. coli* BL21 (DE3) cells, which were then cultured in LB medium containing kanamycin (30 mg/L) at 37 °C. When D_{600} reached ~ 0.6, protein over-expression was then induced using 0.2 mM IPTG at 16°C overnight. The cultures were harvested by

centrifugation (1,500 g, 30 min, 4°C) and the cells were suspended in PBS. After sonication, the resulting lysates were clarified by centrifugation at 16,000 g for 10 min.

For the CBD1^{Sbp9}, the protein was in the insoluble cell pellet, which was washed by PBS buffer and 1 M urea, the resulting cell pellet was then solubilized in 20 mM Tris–HCl pH 8.0 binding buffer containing 5 mM imidazole, 8 M urea were purified with a Ni^{2+}-NTA agarose and eluted with 20 mM Tris–HCl pH 8.0 elution buffer containing 500 mM imidazole, 8 M urea. CBD1^{Sbp9} was refolded using a Sephacryl S-100 column (GE Healthcare, Chicago, Illinois, U.S.) eluted in 20 mM Tris-HCl pH 8.5. Preparation of $EGFL_4$ was similar to the CBD1^{Sbp9} except the Ni^{2+}-NTA was not applied.

The recombinant protein pure was evaluated by 15% SDS-PAGE (Figure S2). The protein concentration was measured by Bradford methods[4].

Supplementary figures

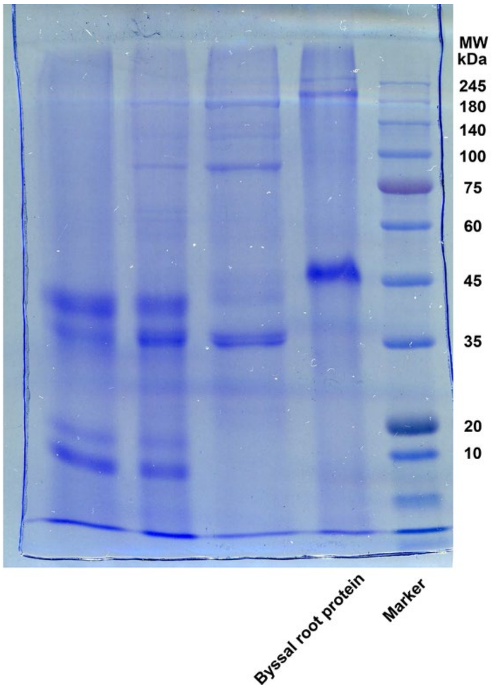

Figure S1. SDS-PAGE of extract from the scallop byssal root

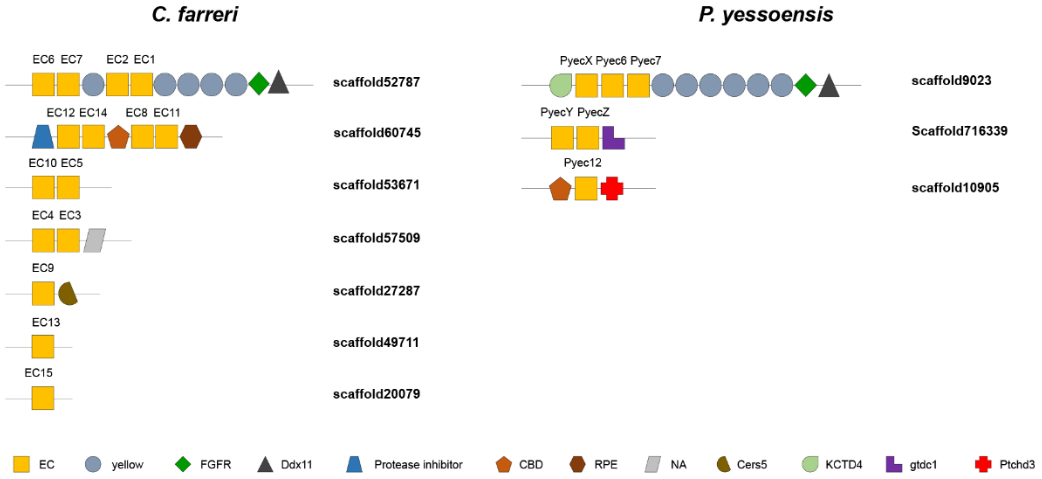

Figure S2. Genomic distribution of EC genes in scallops (*C. farreri* and *P. yessoensis*).

```
79-115       PCYDRCGPNSECDVETFICSCKKNMVGNPYSREGCDV
116-150      PCGGKCIANAFCNEVTNQCECKAGYKGSATTKCYI..
151-185      PCDNKCKENAFCLESENKCQCNPGYQGNPLKGCEL..
186-219      PCGGSCPINSYCKDNKCVCNEGFVGNPYKDGCDV...
220-254      PCGGKCVFNAYCNSKDNKCYCKEGLVGNPYTRCAK..
255-289      PCNDECRQNAYCNKKDNKCYCNKGFQGDPRVGCRK..
290-323      PCDDRCVKDAFCDTDNKCKCKKGLVGDGYIKCGK...
324-357      PCDGKCKKFASCGADNVCHCNPGYVGDPYKGCDV...
358-390      PCKGKCKENARCENEECVCLQGFVGDPYKGCSK....
504-537      PCGGSCKANEHCDMHSQECVCNTGYKLYKKACVL...
538-571      PCGGPCKQYERCDEGSNKCVCMTGYSLFKGSCVV...
572-605      PCGGPCGPNAYCDKNKNQCNCNKGYFTYHGVCAL...
606-639      PCGGPCKQNANCDKNSNQCVCNKGYKEIGGVCAV...
640-673      PCGGKCPQNSRCDEVINECVCLKGYVFFSGSCKV...
674-707      PCGGPCGKNAYCDRISNKCVCNTGYISYKGSCAL...
708-741      PCGGPCKSNEYCNRNANKCECNQGFYVFKGSCVL...
742-775      PCGGPCGLFASCDKSKNQCVCDSGYFLYHGACTL...
776-809      PCGGRCKPNSYCDKSSNQCVCNKGYVNYHDSCVI...
810-843      PCGGPCKSNSYCDQTMNQCVCNKGYIQYHGSCTL...
844-877      PCGGPCEKFAYCDRISNQCVCNQGYKLFKGSCVV...
878-911      PCGGPCGQNSQCDFVTNKCVCYPGYESYKGSCII...
912-945      PCGGPCGSYATCNKVINKCECIKGYKLYHGQCLI...
946-979      PCGGPCGAFATCDEGSNKCVCNKGYFLYNGACSL...
980-1015     PCGGKCPDNAYCNDIANRCQCKQGFFGDAYQGGCHR.
1131-1165    PCGGQCGVHAHCDMLTQECVCDAGYFSFNRGPCAL..
1166-1199    PCGGKCGANAYCDRQANRCVCNTGYRLYQGSCAI...
1200-1233    PCGGECGPNSRCDYLSNKCVCFPGYFLFKGACAL...
1234-1267    PCGGQCAPNSRCDRLTNECVCNTGYFSFHGSCVL...
1268-1301    PCGGHCGPYSYCDKTRNQCVCNTGYFLYHGSCTL...
1302-1335    PCGGKCGPNSRCDRTTNQCVCNTGYFLFQGSCVV...
1336-1369    PCGGRCGNNAFCDKSRNQCVCNTGYFLFQGSCVV...
1370-1403    PCGGRCGPNSVCDKTRNQCVCNTGYFLYHGSCTL...
1404-1437    PCGGQCAPNSRCDRTTNQCVCNTGYYSFHGSCVV...
1438-1471    PCGGRCGPNSVCDKTRNQCVCNTGYFLYHGSCTL...
1472-1505    PCGGKCGPNSRCDRTTNQCVCNTGYFSFQGSCVV...
1506-1539    PCGGRCGNNAYCDKSRNQCVCNTGYFLYHGSCTL...
1540-1573    PCGGNCRPNSRCDRTTNQCVCNTGYFSFQGSCVL...
1574-1607    PCGGHCGPNSECDKTRNQCVCKSGYYLFSGSCVV...
1608-1641    PCGGHCGPNSQCDKTRNQCVCNTGYFLYHGACAL...
1642-1675    PCGGKCAPNSHCDRTSNQCVCNTGYYSFHGSCVV...
1676-1709    PCGGRCGPNSECDKTRNQCVCNTGYFLYHGSCTL...
1710-1743    PCGGKCGPNSRCDRTTNQCVCNTGYFLFQGSCVV...
1744-1777    PCGGRCGNNAYCDKSRNQCVCNTGYFLYHGSCTL...
1778-1811    PCGGPCRPNSRCDRTTNQCVCNTGYFSFHGSCVV...
1812-1845    PCGGRCGNNAYCDKSRNQCVCNTGYFLFQGSCTL...
1846-1879    PCGGNCGYNAYCDKVRNQCVCNSGYVLFRGSCTL...
1880-1914    PCGGRCGQNEQCDSLSNQCVCKTGFIKFHGGPCQL..
1915-1949    PCGGPCGAHSYCNQGTNQCTCDVGYFKFQGGACAL..
1950-1984    PCGGKCVYNAFCDKGTNTCKCNPGLVGDGSKKCGI..
```

Figure S3. Sequence alignments of EGFLs in Sbp9

The PCGGPC motif at the first two Cys residues, which is unique among other EGFLs (highlighted by the blue in 2D), is highlighted by the blue.

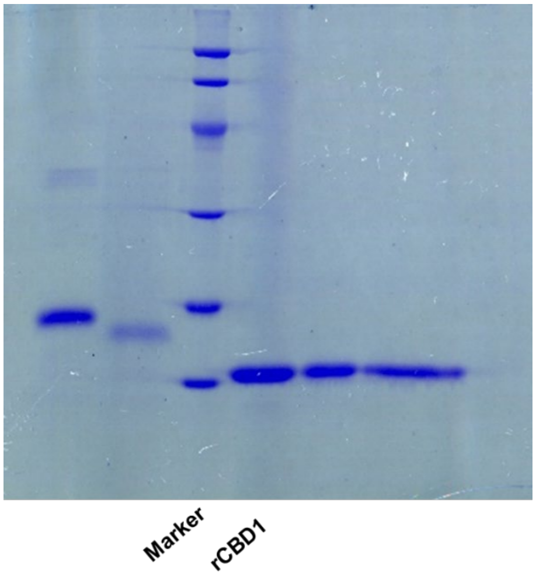

Figure S4. SDS-PAGE of purified rCBD1

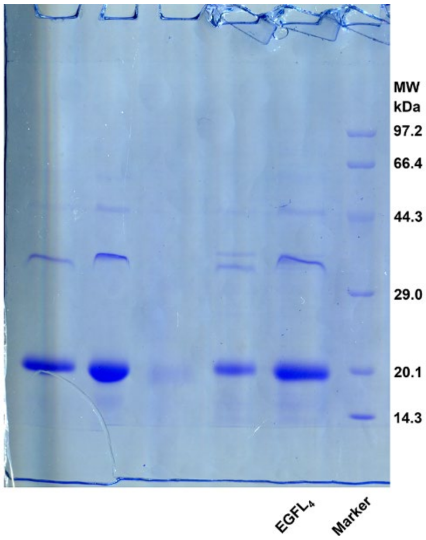

Figure S5. Analyses of recombinant EGFL$_4$ by SDS-PAGE.

Supplementary Tables:

Table S1 Annotation of the proteins identified by MS analysis of band 1 extraction from the byssus root

Gene ID	Unique Peptides	Score	E value	Annotation	Foot specific/ over	Relative intensity (%)
52787.5	38	94	1.00E-17	Tenascin-X	specific	77.3
47691.8	54	93.6	2.00E-18	Matrilin-3	specific	12.7
51689.11	17	582	0	CD109 antigen	Specific	5.2
51689.10	5	592	0	CD109 antigen	specific	
61191.54	13	756	0	SCO-spondin		
341.8	19	3685	0	Myosin heavy chain, striated muscle		
47691.7	4	#N/A	#N/A	#N/A		
13991.1	2	705	0	SCO-spondin	specific	
54635.5	2	#N/A	#N/A	#N/A		
59679.8	6	1067	0	Paramyosin		
48907.12	2	#N/A	#N/A	#N/A	specific	
64771.1.1	2	#N/A	#N/A	#N/A	over	

Table S2 Annotation of the proteins identified by MS analysis of band 2 extraction from the byssus root

Gene ID	Unique Peptides	Score	E value	Annotation	Foot specific/over	Relative intensity (%)
47691.7	11	#N/A	#N/A	#N/A		52.9
48907.12	15	#N/A	#N/A	#N/A	specific	33.7
52787.5	14	94	1.00E-17	Tenascin-X	specific	9.7
47691.8	6	93.6	2.00E-18	Matrilin-3	specific	
55319.24	2	167	1.00E-47	Calponin-3		
30077.9	3	#N/A	#N/A	#N/A	specific	
61295.39	3	164	8.00E-41	Collagen alpha-1 (XII) chain	over	
61191.54	4	756	0	SCO-spondin		
24253.2	2	409	10.00E-130	Beta-hexosaminidase subunit beta		
53111.39.1	2	512	1.00E-168	60 kDa neurofilament protein		
54475.1	2	348	1.00E-107	Peroxidase-like protein	over	
59679.8	2	1067	0	Paramyosin		
64139.6	2	894	0	Tubulin alpha chain, testis-specific		
42195.8	3	924	0	Tubulin beta-4B chain		
14907.6	6	779	0	Actin-2		

Table S3 Summary of EGF/CBD domain comparison between Sbp9 and other related proteins (TNL/NCX1).

Query	Subject	Score	Expect	Identities	Positives	Gaps
Sbp9(EGFL5)	TNL (EGFL3)	48.9 bits(115)	5.00E-16	22/33(67%)	24/33(72%)	0/33(0%)
Sbp9(CBD)	NCX1 (CBD2)	86.7 bits(213)	2.00E-28	43/114(38%)	70/114(61%)	2/114(1%)

Table S4 Number of free thiol of $EGFL_4$

Name	Number of free thiol
$EGFL_4$	1.71
Lysozyme	0

Table S5 Conformational change of byssal proteins

Gpoup	Secondary structrue			
	β-sheet	Coil	α-helix	β-turn
Control	32.31	36.58	21.67	9.44
DTT/EDTA	51.00	26.56	13.80	8.64
DTT/EDTA treated and then $EGFL_4$ added	26.47	39.16	24.23	10.14
DTT/EDTA treated and then BSA added	45.17	26.90	18.27	9.66
DTT/EDTA treated and then Ca^{2+} added	48.35	29.30	15.50	6.85
DTT/EDTA treated then $EGFL_4$ and then Ca^{2+} added	36.81	34.79	19.50	8.90

Table S6 The primers used in this study.

Name	Forward primer (5'-3')	Reverse primer (5'-3')
CBD1^{Sbp-9}	**CGGGATCC**AGCAAACCGAG TACCTTTAGCC[a]	**CCGCTCGAG**TTAGTCGTCAT TTGCAATGTTAATGG[a]
$EGFL_4$	**CGGGATCC**GCACCGTGCGG CGGTAGTTG[a]	**CCGCTCGAG**TTAAACAACGC AAGAGCCTTTAAAC[a]

a. **Bold red** are restriction enzyme sites and the words shown **bold** are bases flanking the recognition sequences.

1 Li, Y. *et al.* Scallop genome reveals molecular adaptations to semi-sessile life and neurotoxins. *Nat Commun* **8**, 1721, doi:10.1038/s41467-017-01927-0 (2017).
2 Robinson, M. D., McCarthy, D. J. & Smyth, G. K. edgeR: a Bioconductor package for differential expression analysis of digital gene expression data. *Bioinformatics* **26**, 139-140, doi:10.1093/bioinformatics/btp616 (2010).
3 Ellman, G. L. Tissue sulfhydryl groups. *Arch Biochem Biophys* **82**, 70-77 (1959).
4 Bradford, M. M. A rapid and sensitive method for the quantitation of microgram quantities of protein utilizing the principle of protein-dye binding. *Anal Biochem* **72**, 248-254 (1976).

Supplementary Data 1

Full length for Sbp9

MAFPKQSKGLSLWICTLTLVASSLADHYASVPYQGGGVIGGGSSGGSKTWCCENANLINGRC
VCKENYKGDSKHPCDQPCYDRCGPNSECDVETFICSCKKNMVGNPYSREGCDVPCGGKCIA
NAFCNEVTNQCECKAGYKGSATTKCYIPCDNKCKENAFCLESENKCQCNPGYQGNPLKGCE
LPCGGSCPINSYCKDNKCVCNEGFVGNPYKDGCDVPCGGKCVFNAYCNSKDNKCYCKEGLV
GNPYTRCAKPCNDECRQNAYCNKKDNKCYCNKGFQGDPRVGCRKPCDDRCVKDAFCDTDN
KCKCKKGLVGDGYIKCGKPCDGKCKKFASCGADNVCHCNPGYVGDPYKGCDVPCKGKCKE
NARCENEECVCLQGFVGDPYKGCSKPSTFSLVQRIYNVKENVGQVQIEVVRSEGSAGSYVVS
WQTSDGSAVQKQDYVGTKGSVNFKSEAKSQKFNIKIVNDKEYEPDESFTVSLVSVSAGGSLG
AITLATINIANDDAPCGGSCKANEHCDMHSQECVCNTGYKLYKKACVLPCGGPCKQYERCDE
GSNKCVCMTGYSLFKGSCVVPCGGPCGPNAYCDKNKNQCNCNKGYFTYHGVCALPCGGPC
KQNANCDKNSNQCVCNKGYKEIGGVCAVPCGGKCPQNSRCDEVINECVCLKGYVFFSGSCK
VPCGGPCGKNAYCDRISNKCVCNTGYISYKGSCALPCGGPCKSNEYCNRNANKCECNQGFY
VFKGSCVLPCGGPCGLFASCDKSKNQCVCDSGYFLYHGACTLPCGGRCKPNSYCDKSSNQCV
CNKGYVNYHDSCVIPCGGPCKSNSYCDQTMNQCVCNKGYIQYHGSCTLPCGGPCEKFAYCD
RISNQCVCNQGYKLFKGSCVVPCGGPCGQNSQCDFVTNKCVCYPGYESYKGSCIIPCGGPCG
SYATCNKVINKCECIKGYKLYHGQCLIPCGGPCGAFATCDEGSNKCVCNKGYFLYNGACSLPC
GGKCPDNAYCNDIANRCQCKQGFFGDAYQGGCHRPTVIQFVRITYEEKESVGQVKIDLERTG
GLYGSVSVTWSTRDISASYGSDFSDNPGLVSFANGVSSASLIINIINDEEYEQTETFSVSLDTVS
VAVGASIGSKNHVTVTIVDDDKPCGGQCGVHAHCDMLTQECVCDAGYFSFNRGPCALPCGG
KCGANAYCDRQANRCVCNTGYRLYQGSCAIPCGGECGPNSRCDYLSNKCVCFPGYFLFKGA
CALPCGGQCAPNSRCDRLTNECVCNTGYFSFHGSCVLPCGGHCGPYSYCDKTRNQCVCNTG
YFLYHGSCTLPCGGKCGPNSRCDRTTNQCVCNTGYFLFQGSCVVPCGGRCGNNAFCDKSRN
QCVCNTGYFLFQGSCVVPCGGRCGPNSVCDKTRNQCVCNTGYFLYHGSCTLPCGGQCAPNS
RCDRTTNQCVCNTGYYSFHGSCVVPCGGRCGPNSVCDKTRNQCVCNTGYFLYHGSCTLPCG
GKCGPNSRCDRTTNQCVCNTGYFSFQGSCVVPCGGRCGNNAYCDKSRNQCVCNTGYFLYHG
SCTLPCGGNCRPNSRCDRTTNQCVCNTGYFSFQGSCVLPCGGHCGPNSECDKTRNQCVCKSG
YYLFSGSCVVPCGGHCGPNSQCDKTRNQCVCNTGYFLYHGACALPCGGKCAPNSHCDRTSN
QCVCNTGYYSFHGSCVVPCGGRCGPNSECDKTRNQCVCNTGYFLYHGSCTLPCGGKCGPNS
RCDRTTNQCVCNTGYFLFQGSCVVPCGGRCGNNAYCDKSRNQCVCNTGYFLYHGSCTLPCG
GPCRPNSRCDRTTNQCVCNTGYFSFHGSCVVPCGGRCGNNAYCDKSRNQCVCNTGYFLFQG
SCTLPCGGNCGYNAYCDKVRNQCVCNSGYVLFRGSCTLPCGGRCGQNEQCDSLSNQCVCKT
GFIKFHGGPCQLPCGGPCGAHSYCNQGTNQCTCDVGYFKFQGGACALPCGGKCVYNAFCDK
GTNTCKCNPGLVGDGSKKCGIPCDGRCGSFKHAHCDHTSNRCVCDVGWKGNPYGSLGCVC
DHSGGGNGHY

Supplementary Data 2

Related protein sequences

>Cf_EC1
MAFPKQSKGLSLWICTLTLVASSLADHYASVPYQGGGVIGGGSSGGTKTWCCENANLINGRC
VCKENYKGDSKHPCDQPCYDRCGPNSECDVETFICSCKKNMVGNPYSREGCDVPCGGKCIA
NAFCNEVTNQCECKAGYKGSATTKCYIPCDNKCKENAFCLESENKCQCNPGYQGNPLKGCE
LPCGGSCPINSYCKDNKCVCNEGFVGNPYKDGCDVPCGGKCVFNAYCNSKDNKCYCKEGLV
GNPYTRCAKPCNDECRQNAYCNKKDNKCYCNKGFQGDPRVGCRKPCDDRCVKDAFCDTDN
KCKCKKGLVGDGYIKCGKPCDGKCKKFASCGADNVCHCNPGYVGDPYKGCDVPCKGKCKE
NARCENEECVCLQGFVGDPYKGCSKPSTFSLVQRIYNVKENVGQVQIEVVRSEGSAGSYVVS
WQTSDGSAVQKQDYVGTKGSVNFKSEAKSQKFNIKIVNDKEYEPDESFTVSLVSVSAGGSLG
AITLATINIANDDAPCGGSCKANEHCDMHSQECVCNTGYKLYKKACVLPCGGPCKQNANCD
KNSNQCVCNKGYKEIGGVCAVPCGGKCPQNSRCDEVINECVCLKGYVFFSGSCKVPCGGPCG
KNAYCDRISNKCVCNTGYISYKGSCALPCGGPCKSNEYCNRNANKCECNQGFYVFKGSCVLP
CGGPCGLFASCDKSKNQCVCDSGYFLYHGACTLPCGGPCEKFAYCDRISNQCVCNQGYKLFK
GSCVVPCGGPCGQNSQCDFVINKCVCYPGYESYKGSCIIPCGGPCGAFATCDEGSNKCVCNK
GYFLYNGACSLPCGGKCPDNAYCNDIANRCQCKQGFFGDAYQGGCHRPTVVQFVRITYEVK
ESVGQVKIDLERTGGLYGSVSVTWSTRDISASYGSDFSDNQGLVSFANGESSASLIINIINDEEY
EQTETFSVSLDTVSVAVGASIGSKNHVTVTIVDDDKPCGGQCGVHAHCDMLTQECVCDTGYF
SFNRGPCALPCGGKCGANAYCDRQANRCVCNTGYRLYQGSCAIPCGGECGPNSRCDYLSNK
CVCFPGYFLFKGACALPCGGQCGPYAYCDKTRNQCVCNTGYFLYHGSCTLPCGGKCGPNSRC
DRTTNQCVCNTGYFLFQGSCVVPCGGQCAPNSHCDRTTNQCVCNTGYYSFHGSCVVPCGGR
CGPNSECDKTRNQCVCNTGYFLYHGSCTLPCGGRCGNNAYCDKSRNQCVCNTGYFLYHGSC
TLPCGGHCGPNSECDKTRNQCVCKSGYYLFSGSCVVPCGGHCGPNSQCDKTRNQCVCNTGY
FLYHGACALPCGGKCAPNSHCDRTSNQCVCNTGYYSFHGSCVVPCGGRCGNNAFCDKTRNQ
CVCNTGYFLYHGSCALPCGGQCAPNSRCDRTTNQCVCNTGYYSFHGSCVVPCGGRCGPNSE
CDKSRNQCVCNTGYFLYHGSCALPCGGKCAPNSRCDRTTNQCVCNTGYYSFHGSCVVPCGG
RCGPNSQCDKSRNQCVCNTGYFLYHGSCALPCGGPCRPNSRCDRTSNQCVCNTGYFSFHGSC
VVPCGGRCGNNAYCDKSRNQCVCNSGYFLFKGSCTLPCGGQCRPNSRCDRTTNQCVCNTGY
FSFHGSCVVPCGGRCGNNAYCDKSRNQCVCNSGYFLFKGSCTLPCGGNCGYNAYCDKVRNQ
CVCNSGYVLFHGSCTLPCGGRCGQNEQCDSLSNQCVCKTGFIKFHGGPCQLPCGGPCGAHSY
CNQGTNQCTCDVGYFQFQGGACALPCGGKCVYNAFCDKGTNTCKCNPGLVGDGSKKCGIPC
DGRCGSFKHAHCDHTSNRCVCDVGWKGNPYGSLGCVCDHSGGGNGHY

>Cf_EC2
MAVSSPMHHFFMICLLVIVSSVVSSATYRYETVQEKAPSPYKDDSGPSPYKDDLGSSPYIDDSG
PSPYKVDSGPSPYKVDSGPSPYKDDSGPSPYKDDSAAKTKCCDNAILINGRCVCKQNYKGND
KHKCIQPCDGKCMNNSYCSEADNKCYCYDGYVGNAYLEGCDVPCGGKCAINAYCRPKDNK
CCCKDGYEGNPYFKCSKPCDDACQNNSYCSKRDNKCYCNNGYIGDPTTGCSLPCDKKCCKD
SYCDEVTNKCVCKKGLIGDPYVSCKQPCDGSCKKNAYCRESDNKCVCNDGYIGDGYQGCDV

PCGGKCKDNTYCNPDSNTCQCDPGFIGDPYVQCYEPCGVQFVQSNYTVDESIKRLSIQVSRK
GGSYGAVTVKWETKDLTATFGNDFSNNNGKITFIKGETIQTFTITIVNNKIHEPTETFSVRLTAIS
AGGFIGFLKVTTITIIDDDEACGGPCGSNAYCDQTSQKCVCNVGYNLYKGSCTVPCGGPCELN
GYCDRSLNTCVCKHGYQIFNGACQLPCGGSCGDYAYCDKDTNQCICNNGYVSYQQACVLPC
WGSCGDNQYCDTDSNECVCNKRYITYHGSCVSPCDGRCVENAYCDQKINKCVCKKGYVLH
YGKCIGPCDNACKENAYCNHENVCKCHSGFYGNPLKGCTLPCGRKCKAHAHCDLEPKQQCN
CDDNYIGNPYVGCKLPCDGSCGKNAYCDEDTNKCVCNKGYFIYGGSCALPCGGPCKKYAHC
DRDSNECVCDKGYTVYDNSCVIPCGGSCGPYAQCSHYTNRCICDYGYVLFQGSCVRPCNNA
CKENAYCSHDNVCKCHAGYYGNPLRGCTLPCDGRCKDYSHCVLKPKPRCVCNDNYIGDPYS
GCTLPSDVGFLRARYSVLESDEEVVLQLTRTGGITESVTVRWLAKSMTATYRKDFDRNKGQV
TFPKGQTMVEFIIGIINDQVYEHPEQFSVSLTTISKGGILGQINTAIVTIRNDDKPCDGQCGINAV
CEMSSQKCICKHGYFLSQGSCILPCGGQCKSNAYCDHGTNRCVCNVGYEMYDGSCAVPCGG
KCGPNSYCNKLSNQCVCFTGYDTYNGTCSLPCDGSCVYNAYCDRDSNTCKCNKGLVGDATK
SCGLPCDGRCRYFPHSHCDDNTNMCACDKEWHGDPYGKEGCICKGENHYGRVPKYSDQTQ
PSYFRNG

>Cf_EC3
MAHLLRTIGLLFIICAVILVAPSCAKRNRKRKNKAVYSPPKVVTPPPKKTTPPPKKTTLPPKKIT
VPPKKVPVPPKGNDYAQSTGGNYPPSTGGDYAPSNGGNYPASTGGDYAPSTGGDYPPSTGGD
YAKGGNYVPAKKCCDNAVLKNGRCVCKPHYYGTSTHPCVRPCWDKCGPNTRCDEETFKCIC
KDGFVGDPYKGCHLPCHGKCVENASCNEETDKCECDEGLVGDGAIECFEPSTVVFAKDSYEV
SESQSTLKVEVLRQGGINSIVTVKFQATDGTAVHGSDYFNNHGVITFQRTVTSQYITIYIHNDLV
FEQSETFKLTLVSVIRGEIGEPSTTTITITDDDDPCGGKCGPHSHCDEATQQCVCDEGFVEDSYH
GGCTLPCGGRCCANAYCNPKDNRCYCNEGYYGIPTLNCYLPCKGACKANAICGKNNQCYCK
TGFYGNPYEGCYPPCHGKCGTNARCDISTHTCVCNDGFFGDPYDHCDKAAVIGIEEPTYSVNE
NAGFVEITVIRTGSTFGTIFAIWKTSDITATSGEDYGGGIGIISFADGQDSTTISIFIANDKIFEGAE
TFSVSLTFVSAGGVLGVHIQTIVTIVDNDEPCGGACPPNKHCDEKTQKCVCNKGYYPDHYGG
CQLPCGGRCCANAFCDYKSNTCKCNTGYIGIPTVACFKPCKGGCGANAYCVNNKCVCKPGY
WGNPYSGCYLPCHNLCKENAECDLTTHTCRCLPGFVGDPLKGCTKPGTFIFTQTTYTVQENV
GIVIITVSRVGSSFGAVKVSWSTKDGTAIQGRDYKHAQGVIWFASGEVSRTISIVIINDNVYEKD
ETFTVSLTSISIGILTSSTVATITIKDDDPSCGGPCPPNSHCDEVSQKCVCNHGYHFDANYGGCIL
PCGGRCCLNAYCDVKYNQCKCNAGFIGIGTVGCYKPCKGLCKVNAYCGKDNTCCCKPGFFG
DPYVGCTEACNGRCKENARCDIPTQTCQCLPGFFGDPEVGCGKPGNFEFERSSYEVSESAGSL
TVTVRRVGSSSGSVAVTWATTDTSATSNKDYFNTQGVLFFANQVITKTITFYINDDSIYEDNESF
DVTLLSVIPAGGLGSIRVTTVTIKSDDPPCGGPCPVNSYCDIPSQKCVCNKGHISFKGSCVIPCG
GKCCANAYCSHKTNQCHCNPGYLGNPTQKCYIPCNNACKGNSYCGKDNKCHCKTGYYGNA
YTECHLPCHGLCKKYSKCNLVTQQCVCLPGYFGDPKVGCHGMFVFVLVQSTYFVSESVTSVE
IQVKRTGSVSGSVQVTWTATDLTAIHGEDYANSGGTLTFNDGDVFKTIIVLIRQDEKFEADEQF
VVTLTSVTTGGVLGLLRKATVTIENDDKPCGGKCVFNAHCDEATDTCICNKGLVGDGTVKCD
YSILYFQIPVADIAQPTHTLTVTTRQTNVSVTTNTEGIPITEDVYALLEDIPEDMEVILVVVMELP
AEVIPVAVMEVPAEVIPVAVTVEDIPAAVMEIPAEVIPVAVIVEVIPVAVMEILVAVIVEDIPVAVTD

PPAEVIPVAVINVPALVSAYAIPVTMVEVIANQALNKIFEINTVCRMITASETSIEPVLVTSGTGSG
NRMERGFE

>Cf_EC4
MAHLLRAVGFFFVINAAILFAPSSGSDYAPVSKQGYVPTHDAHLPPPGDYAPPKGGDYPAQKC
CENAKLINGRCVCKPDYYGPSTHPCVQPCWDKCGPNTRCDVNSFKCYCKDGFIGDPYKGCH
VPCYGKCVENASCNLDTDECECNDGLVGDGTVKCFEPSTFVFEKETDTVSESQSTLKIGVLRQ
GGTDGVVAVVFKTTDGTAIHGSDYFNSHGVIIFHDQVAIQYITIYINNDLVFELSETFTVTLVSV
SIGNIGEPSTTTITITDTDDPCGGKCGPHSYCDEEAKQCRCDKGYVEDSYHGGCKLPCGGRCC
ANAYCNPKDNRCYCNEGYHGIPTLNCYQPCKGACKANAICGKNNICVCKPGFFGNPYEGCYP
PCHGKCGTNARCDIPTHTCVCNDGFFGDPYDHCDKAAVIGIEHPTYSVNENAGVVEITVIRTG
STFGTIYAIWKTSDITATSGEDYGGGIGIISFAAGQDSTTISIFIASDKIFEADETFSVSLTFVSAGG
VLGVHIQTIVTIVDDDEPCGGPCPSNKHCDEKAQKCVCNKGYYPDRYGGCQLPCGGRCCAN
AFCDYKSNTCKCNTGYIGIPTVACFKPCKGSCGTNAYCVNNKCVCKPGYWGNPYSGCYLPC
HNLCKENAECDLTTHTCRCRPGYWGDPLVGCTKPGSFIFTQTTYTVQESVGIVSITVSRVGSSF
GAVKVTWSTKDGTAIQGSDYKHAQGVIWFASGEVSRTISIVIINDNVHEKDETFTVSLTSISIGIL
TSSTVATITIQDDDPSCGGPCPPHSHCDEVSQKCVCDHGYHKDPHYGGCVLPCGGRCCVNAY
CDVRYNQCKCNAGFIGIGTVGCYKPCKGLCKVNAYCGKDNRCCCKPGFFGDPYVGCHEACH
GRCKANTRCDPHTHTCHCLPGFYGDPLVGCGKPGRFEFERASYETSESSGSLTVTVRRIESSSG
AVAVSWATTDVSAIHDVDYYNNQGVLFFADGVTIRTITFTIDDDSIYEENEIFRVSLLSVVPAGG
FGTITTTTVTIKSDDPACGGPCPLNSYCDIPSQKCICKKGHILFHGSCVIPCGGKCCANAYCSHK
DNQCHCLSGYIGNPTQKCYIPCNNACKGYSYCGKDNKCHCKTGYYGNAYLECHLPCHGLCK
KYSKCNLVTQQCVCLPGYFGDPKVGCHGMFTFTLVQSSYSVSESVTSVKIQVKRTGSVFGSV
KVTWTATDLTAIHGQDYANSGGILYFKQGDVFQTITVLILNDEKFEADEQFVVTLTSVTTGGVL
GLLRKATVTIENDDKPCGGKCVHNAHCDEASDKCICNKGLVGDGTVKCDCKF

>Cf_EC5
MVDVEPMLDVISQSIHVFVMMVSLVTHMLIVTIFEAEETFSVSLTHVSVGGVLGVHIQTIVTIV
DNDEPCGGACPPNKHCDEKTQKCVCNKGYFSDHYGGCQLPCGGRCCANAFCDYKSNICKC
NTGYIGIPTVACFKPCKGGCGTNAYCVNNKCVCKPGHWGNPYSGCYLPCHNLCKENAECDL
TTHTCRCRPGYWGDPLEGCTKPGSFIFTQTTYTVQESVGIVSITVSRVGSSFGAVKVTWSTKD
GTAIHGSDFVHIQGVIWFASGEVSRTISVVIINDNVYEKVETFTVSLTSISIGILTSSTVATITIKDD
DPSCGGPCPPHSHCDEVSQKCVCDHGYHKDPHYGGCVLPCGGRCCVNAYCDAHYNQCKCN
AGFIGIGTVGCYKPCKGLCKVNAYCGKDNRCCCKPGFFGDPYFGCHEACHGRCKANTRCDP
HTHTCHCLPGFYGDPLVGCGKPGRFEFVQSNYEVSESSLSVTVTVRRIESSSGSVAVTWATTDV
SATHDVDYYNTQGVLVFANGVITRTITFTIEDDSIYEENETFTVSLLSVVPAGGFGTRTTTTVTI
KSEDPACGGPCPLNSFCDIPSQKCICKKGHIFFHGSCVIPCGGKCCANSYCSHKDNQCHCNSG
YIGNPTQKCYIPCNNACKGNSYCGKDNKCHCKTGYYGNAYLECHLPCHGLCKKYSKCNLVT
QQCVCLPGYFGDPKVGCHEPSVFVLVQSSYSVSESVTSGEIQVKRTGSVSGSVRVTWTATDLT
AIHGEDYANSGGTLTFNNGDVFKTIIIIIRNDE

>Cf_EC6
MNDVTNPVTSVSVSTDILNSADLVFVIPCGGSCPPNSKCSSSDNRCHCNPGFLGDPYKGGCHR
RSVVEFAITTYTVSESAGTVSLQLSRSVGLFGSVTVSWSTRDGSARYPGDFSQNSGVATFGNG
QDSTTFSIAIVNDQIYESSESFTVTLSVAASSSSVVTIGQRSVATVTITNDDPPCGGPCGPNARCD
LSSQKCVCNSGYFRFGGSSTCQLPCGGPCRGPNERCDEPSDQCVCIDRYIKFHGACVLPCGGP
CRSNERCDIPSDQCVCIERYVKFHGACVLPCGGSCPLNSRCSSSDNRCHCNPGFLGDPYKGGC
HRRSVVEFAITTYTVSESAGTVSLQLSRTVGLFGSVTVSWSTRDGSARYPGDFSQNSGVATFG
NGQSSTTFSIAIVNDQVYESSESFTVTLSVAASSSSVVTIGQRSVATVTITNDDPPCGGPCGPNA
RCDLTSQKCVCNSGYFRFGGSTCQLISHVDLVLLEPFVLMDMSFLVEDVVHQIQNVTDKPTN
VNVLEVTKEIHTRVDVDCLAEDHVEDLMKDVTNPVTNVSVSTDILNSTELVFVIPCGGPCPSN
SKCNLAVNRCQCVPPYKGDAHHGGCKLPCGGNCPPNSKCNLAVNRCECIPPYKGDAHHGGC
KLPCGGPCPKNSQCSSSDNRCHCNHGFEGNPYHGGCHLRSVVEFGQIIYTVSETAGQVSLQLR
RTGGLSTSVTVSWSTQNGIAGNGGSAVYPDDFQQNEGHATFASGQIETTFNIGIVNDQLYEQPE
AFTVTLSVTQGSNTNIVEIGQRSVATVTIISDDPPCGGKCVFNAYCDKESNTCRCNHGLVGDPT
KKCGIPCDGRCGSFPHSHCDKITNRCICDTGYTGNPYGSVGCVCAGGHGGGGGGGYSGGSSG
ISYHGGVSGGSYHGGSSGGSYQGGSTGGSYSGGSSGGSYHAGSSGGGGYEPPHGSDY

>Cf_EC7
MAIRKLSQGYCLWICVLLAATYADHVSDYGFGGGYSGGNTGGSYGGGNTGGSYGSGNTGGS
YSAGNTGGSYSGSSHVDVGGYDDNSHVSGGGYGGASYGGGGVVIVDHSHTGGGYGGSGGD
KTHCCDNADLINGRCVCKENYKGHSTHPCDKPCYGRCGKNAKCNKQTFECYCFEGYIGDPY
TVCDLPCGGKCGTNAYCDSGSNSCRCKTGFIGDAKTRCSEPSVVSFAQSSYSVRENSGVLKIK
VIRKGGTFGSFTVQWTVSDGSATSPSDFSFTLAPLQFNNGDGVQIISINIRNDEFFEPDESFTITL
TSVTAEGRIGSPDVTTVTIINDDKKCGGKCKANAYCRESDNTCVCNTGYIGDPTKGCKRPCDG
KCKKNSFCDPQDNRCKCKHGMVGNPYKEGCDVPCGGKCVLNAFCRERDNKCYCKEGYIGD
GTVKCAKPCNDECRHNAYCSKKDNTCYCNKGYIGDPKVGCDLPCGGKCASTAYCDSGSNTC
KCKKGLVGDPHVRCGKPCDGKCKKNAFCNDNNECQCNPGYVGNPLVGCDRPCGGKCRTNA
FCDEHSNTCKCNRGYIGDPQRGCYEPSVFQFVQRLYTVSEDDGSVNIEVVRSKGTEGSYTVS
WEATDGSARFPSDYGNSKGTVVFKPNEKSDKISIKIVNDKVFEPTESFTVTLTAVSANGALGVL
TVTTVTITNDDPECGGPCGANAHCDSPTQRCVCNVGYRLIRGSCALPCGGPCGPNAQCNERT
NKCECITGYSLIQGSCSRKFQFHCVTGLQKSCFSQSKIYHNDKKKSSSTIKLRKLKSKKHPNCK
LLTSTELQDHAEDHADQTSDVTLDQTNAFVSKDTDYSTEVVSFHVEDVVHQIQNVAVQTTDA
IVIQGLKEIHITEDVNVRIGFHNDFVKQVS

>Cf_EC8
MFYQLPTFSLLLAVCVVSIITQTHGGYVAPPVKTCCENAYPSYGRCICKNGYHGDSNHPCVPP
CNGKCGKNTYCDLKWFKCFCKPGFKGDPYEGCCLPCGGDCIANAFCDTLSDKCKCNDTFIG
DPKVNCFKPSVCHFVRTQYVVSETAGFVRIQVNRINGIYGTVTVRWVTTSITATLNADYIGAS
GSITFLTGEATKFIQIKIVNDNIYERNETFAVVLTSITIGHRIGVPCKTVVTIISEDKPCGGYCKKN
SHCDIPTQKCVCNTGYYLWHGQCELPCYGECCKYAKCNIQTFKCCCIPGYYGDPTKQCAKPG
IFEIERREYTVKESAIEVLIVVKRTGSFFGNVKVTWVAKDITAIHGHDYVNHGGTIFFASGVTSQ

IIKVKILNDVIYEKREQFSVRLVTISSGGRFGAIVVTTVNIMDDDEPCHGHCSKFHAHCNEFTQ
KCECDIGFIGNPYECCKKPCDGKCLYNAKCDKDHNVCVCNPPLVGDGVTKCEPPCGGPCPVN
SHSHCDKKINKCVCDDHYFGDPFHGGCKCQTDYKG

>Cf_EC9
MLQLPTVSLFFVASSVLLLIGTSQGTYLPPAKACCDNARLINGRCVCKDNYYGDSTHKCRQPC
DGKCDKNEKCDLYTFICSCKPGFVKLPNSDKCVVPCGGDCITHAQCNKNINKCECTKPYIGD
ASVACYLPSKFQLKRPAYIVSEDVGAVKIQVDRIEGVIGAYDVKWQTNDVSAKLNLDYKFKT
GTVTFGNLQTTGFISIPIIDDTIYEVDEKFELTLVAVTNGGKLGTLIKTVVTIHDNDLPCGGKCK
RNSYCHIPSQKCVCNPKFVLYRGNCEKPCFDRCCLNAYCNEKEFKCYCKEGFYGQPTKRCEK
PSEYKLRDEEYTVKESSLVVKIVVLRTVSTLGPAKVTWVATDVSAKHGLDYIGHKGDIYFKDG
QSSATFTIEIVNDKFYEEKEKFFVTLTTVTAPVPCNGRCVTNARCDPVKKVCVCLPGYIGNAY
KECHRPCHGECLKNAYCDEEVNHCVCNKGLVGDGVKKCAPPCDDRCKNFKHSHCDYKSNR
CVCDDTYHGDAYTNSCVCGTQHY

>Cf_EC10
MTHLLRTIGVFFVVYAAIFVAPSEGGRKYRKGKYVPPPKVYVPPPKVYVPPKDDYVPPPNND
YVPFKKDTYVPPPKVYVPPKDDYGPPPKNDYVPFKKVYVPPPKVYVPPKDDYVPPPKNDYV
PFKKVYVPPPKVYVPPKDDYVPPPKNDYVPFKKVYVPPPKVYVPPKDDYVPPPKNDYVPFKK
VYVPPPKVYVPPKDDYVPPPKNDYVPFKKVYVPPPKVYVPPKDDYVPPPKNDYVPFKKVYV
PPPKVYVPPKDDYVPPPKNDYVPFKKVYVPPPKVYVPPKDDYVPPPKNDYVPFKKVYVPPPK
VYVPPKDDYVPPPKNDYVPFKKVYVPPPKVYVPPKDDYVPPPKNDYVPPKKVYVPPKDNYK
PPPKDDYVPLKDDYGPPKDDYFPSKGGDYNDGGYGPSSGGGYVPAKKCCENAVLINGRCVC
KPNYYGSSTHPCVKPCWNKCGDNARCDVKEFKCYCKDGFIGDPEKGCHVPCHGKCVENAS
CNEETDTCECNEGLIGDGILGCFEPSTVQFGRESYEVSESVRTLKVEVLRQGGIDSVVSVVFKA
TDGTAIHGSDYFNNHGTIIFLKGEQTQYITIYIINDSVFEQSETFQLTLVSVIRGEIGVPSTTTITIT
DNDDACGGKCGSHSDCDEATRQCRCHKGYVEDPYHGGCTLPCGGRCCANAYCNPKDNRCY
CNEGYYGIPTLNCYKPCKGACKANALCGKNNQCFCKTGFYGDPYVGCDHIQTDDEFSFTSLC
NWEKVDPLLLYDFRCFNYSHGCPFLVVHGREVDIADDS

>Cf_EC11
MAKYSPNQIQHNRLETPMPILVTFCVPCGGECSTTAYCDETTNQCVCNPGLVGNPKRGCARPC
NDACGENAFCNKDDNKCYCDDGYIGNPTAGCTLPCNGKCVENAFCKREQCFCNKGLIGDPT
VQCYVPSVVQFVQTSYTVQENIGQLTVEVRRVKGSAGRFTVAYSTNDKSAKSGEDYTAANGV
LIFNSGVISQSIYIQITDDDEYEPKDETFEVRLSDATGGAKIGELKKAVVTIESKDCTNFEI

>Cf_EC12
MDCYIFPRGKSYEEDFLIMYQRQEEKIEHENRAYTTMKRTGGKFKENSKKSSTLFKMMHQIPT
LGLCFTLLCVLSFLGTTKGTYMPVHCCTGSTPYGSRCVCNPGVHPDRYHNCIEPCWNRCGAN
ADCNKYTFKCFCKSGFVGDPEKGCTRPVSTFQFARSSYTVNENAGFAKLQIDRISGVSGRVTL
SWKSFSISATLNSDYKGASGTITFESNEKWKSIQIPIVNDKLFEKTETFKVILTSASSGGRLGPRR

LTVVSIRDDDLPAKFQFARSSYVVNENVGSVKLQIDRISGGLNKVTLYWKSTGITATLNSDYK
GASGSITFGVGEKWKSIVIPIVNDQIFENTETFKVTLTSASAGGELGPRRETVVTIKDNDPPSKF
QFARSSYAVGESAGVVKLQVDRISGGLQKVTLYWKSTGITATLNSDYQGASGSVTFGVGEKW
KLIQIPIVNDQIFEQIETFKVTLTSASTGGAIGPRSTTVVTIKDDDLPCQGKCKYYAYSYCNPKT
NRCVCKPNYYGDPYHGGCRCGGGGGGY

>Cf_EC13
MLQLPTVSLFFVASSVLLLIGTSQGTYLPPVKACCDNARLINGRCVCKDNYYGDSTHKCRQPC
DGKCDKNEKCDLYTFICSCKPGFVKLPNSDKCVVPCGGDCITHAQCNKNSNKCECTKPYIGD
ASVACYLPSKFQLKRPAYIVSEDVGAVKIQVDRIEGVIGAFDVKWETNDISAKLNLDYKFKTG
TVTFGNLQTTGFISIPIIDDTIYEVDEKFELTLVAVTNGGKLGTLIKTVVTIHDNDLPCGGKCKR
NSYCHIPSQKCVCNPKFVLYRDNCE

>Cf_EC14
MFQPIPAVGLCVTLCVLSLLETTQGSYPVCCKGSKPINNRCVCDEGVHPDKYHNCVEPCWKK
CRANADCNRNTFKCYCKPGFVGNPYVGCLRIAKFQFARSSYVVRENTGIVKLQVDRISGGLH
RVVVKWAATKITAQPGTDYIGNFGTVTFEKGEKWKFIEIRIVNDQIAEPTEKFKVTLRSASPGG
AIGSRSVTTVTITDSDSIAKFQFARSSYVVRENTGIVKLQVDRISGGLHRVVVKWAATKITAQP
GTDYIGNFGTVTFEKGEKWKFIEIRIVNDQIAEPTEKFKVTLTSASPAAAIGSRSVTTYHVEESA
RDTLTPSVTRTPTNVFVKKATTEMRTMEDANVVEGVVISRPNRTMC

>Cf_EC15
MTHLLRTIGVFFVVYAAIFVAPSEGGKRNKKAKYVPPRKVYEPPKDDYRPPPKNDYVPSKKV
YVPSNDDYGSLKDDYVPSKGGDYNDGGYGPSSGGGYVPAKKCCENAELINGRCVCKPNYY
GPSTHPCVKPCWDKCGDNARCDVNEFKCYCKDGFVGDPYKGCHEPSTVKFARESYEVSESV
RTLKVEVLRQGGINSVVSVVFKATDGTAIHGSDYFNNHGTIVFLKGEQTQYITIYIINDSVSYFR
FRTMVLVWHL

>Cf_TNL
MSSSVLFVAFSLFLGAPKTLAWEGCIGQCPEHSWCDVTDNRWKCSPGYTGDPYCSTGCVQNP
ILPQCCENAFRNGSNCVCKSGYVGDGFYSCKPVPCFGRCETNAYCKIVDDKCYCKPGFVGDP
YKGCTVPCDDKCKTNAFCKDSENKCYCKPGFVGDPYEGCKEPCDGKCLTNAYCKASDNKC
YCKPGFTGNPYEGCIGTYFVSVPCGGKCKANSYCRTSDNKCICKSGFTGNPYSGCIVPCWGK
CKANSYCRTSDNKCICKSGFTGNPYSGCIVPCWGKCKANSYCRTSDNKCICKSGFTGNPYSGC
IVPCWGKCKANSYCRTSDNKCVCKSGFTGNPYSGCIVPCWGKCKANSYCRTSDNKCVCKSG
FTGNPYSGCIVPCWGKCKANSYCRTSDNKCVCKTGFSGNPYVGCIRKVPCWGKCRANSYCR
TSDDKCVCKSGFTGNPYVGCIRIVPCWGKCRAYSYCRTSDNKCICKPGYTGNPYVGCTRPVP
CGGKCRANSYCRTSDNKCICRPGYTGNPYYGCTRTVPCGGRCKANSFCRTSDNRCCCRAGYS
GNPYVGCTRTVTVPCGGRCRANSFCRTSDNRCCCRAGYRGNPYVGCTRTVTVKQQKLPERIV
NVSACYSDPNAIRANCSSTEMIAVDKVNAGAKLITTSCMSAASIVNLTNPDCCERDKDNDCYT
PFVNQNANLTYTYHEQCIGQETCGPIPVQWMRIPDSCKNSAQYRNYTGYLDIYHYCIKNTSV

GVLPDVSLTSGSNSALYLQGQQYPAYMPQSRSVNCSLEVIGGCGVSIQAIHISFDENGGVCQQ
NIMISDLSNRTENITCDNNNNYNITKMYSTASNYVTLNISSQRGNAQFWIGFIPNSGGTMDLSC
PAVPRSMGTPCAVTDKPSSETTTDDTTDASTDANTYDSTETSSGSTSIIIAVVVSVILLLIIFIVVL
LLRWKYRKQKEAKVVPEKDDNVSHNGDTDSNHAIDVFRSIPFETSTAPWATAVVGSRGSNLPP
IKSQTNDVPEQDGHKKKKKKKKRKKKMKNNDDEGQILAFEDVGESNE

>Cf_NCX1
MSCDLSDYKCADKGLILPLTSEYTWSIGFRAFVYIVGLLYCFMGVSIIADIFMQAIETITSRSARI
KMPDPSNETGYTEVEVKTWNGTVANLTLMALGSSAPEILLSIIEIVGNGFQSGPLGPSTIVGSA
AFNLMCITGVCIISIPDGIVRRIKNIKVFAVTSIFSIFAYIWLAIILMVTTPDFVDLWEAIITLLLFPI
MVLLAYIADKDYCGKKPIDDESKLLEIDEENLLNGDRADKHVIVEILRRLKREKEATPEDMAR
LTAHLMEQNQSHGRGWYRINAIRNLSGGTKLTTTMTDKTNEDQMGSTASFTSMSEGGKKAII
EFAAPSTAVFEKDGHARITVMRHGNLKNRVLYRVETIDGTAVEGEDYTAFKDTLVFEPNETVK
HIDIQIIDDNIWEPDEVFFGRITLDSEKQNAVVGRRAITQIVILNDDDPGVLEFEHPSFLFKESVG
TALVPVNRTDGADGKVTVTWKTKDMTAIHGRDYENTEGTLTFDHGERTKFIEIQINDDKEFEK
DENFEINLLDTTGGAKIGKLKRTVVTIVNDDEFSGLVSRITSLTNANLDSLRLQKQTWGQQFV
EAMNVNGGDLETATTFDYVMHFLTFGWKLIFALVPPATIWGGWFCFVVSLAMIGLLTAFIGDL
ASIFGCLIGLDDSVVAITFVALGTSLPDLFASRTAALNEKYADTAIGNVTGSNAVNVFLGLGLP
WTMAAVYWSARGTTFEVPAGALGFSVVVFTLCSLLTISFLLLRRNLNLFGSAELGGSKVSKIL
SGLFCIIVWVVYVLLASLNAYEHIPGF

>Py_ecZ
MAHLLRTIGLLFVIYAVILVAPSCAKRNRKGRKSKEGYVPPKKVPVPPTVVHVPPKEVPVPPKK
VPVPPTVVYVPPKEVPVPPKVVYVPPKEVPVPPKEVYVPPKGGDYAPSTGGDYAPPAGDDYA
PPKGGDYVPAKKCCENAVLTNGRCVCKPNYYGSSTHPCVQPCWDKCGINTRCDVNTFKCYC
KDGFIGDPSKGCHVPCHGKCVENASCNPDTDKCECNEGLVGDGTVRCFEPSKIVFAKETYTV
SESVSTLTVEVLRQGGTIGVVAVTFKATDGTAIHGADFFNSHGTIVFGEGVSTQYITIYIVDDSIY
EQSETCDLTLVSVSVGGEIGEPSTTTITITDNDDACGGKCGPHSHCDVPIQQCHCDKGYVEDPY
HGGCTLPCGGRCCTNAYCNTKDNRCYCNEGYYGTPTVNCYQPCKGACKANAICGKDNKCY
CKTGFYGNPYEGCYPPCRGLCGTNARCDISTQTCVCNGGFFGNPLEHCDKAAVIGIAQATYSV
NENAGFVVITVTRTGSTFGTIFASWKTSDITATHGNDYGGGEGVIRFVDGEESTTIRIFIVNDKI
YETDETFSVSLTHVSVGGVLGIHIQTVVTIVDDDEPCGGPCPPNTHCDEKTQTCVCNKGYISD
HYGGCQLPCGGRCCTNAHCDYHDNTCKCNTGYIGIPTVACNLPCKGACKANAYCASNRCVC
KTGYYGNPYEGCYLPCRNLCKGNAVCDLSTHTCRCIQGYWGDPLKGCTKPGSFVFAQTTYT
VQENVGIVSITVNRVGSSFGAVKVTWATKDGSAVHGNDFVNTAGAIWFASGEVSRTISIFIISDN
IFEKEESFTVSLTSISIGTLTTSTVATIIIKDDDPSCGGPCPAHSHCDVVSQKCVCDHGYHLDANY
GGCILPCGGRCCANAYCDAHYNQCKCNTGFIGIGTIGCYKPCKGACKLNAYCGKDNRCCCKP
GFYGDPYDGCTEACKGLCKENTRCDIPTQTCQCLPGFYGDPLMGCGKPGQFVFAKSSYSVTE
GAGSVTVTVNRIGSSSGGVAVTWATTDISATSLVDYFNTQGVLFFADGVITKTITFTIYADKVYE
VDEQFQVTLLSVVPAGGLGSLTITTVTIINDDAACGGPCPVNSYCDKISQKCICNKGYILFHGS
CVIPCGGKCCANAYCSHKDNQCHCNSGYIGTPTQKCYIPCNNACTDYAYCGKDNKCHCKTG

YYGNPYSKCHLPCHDLCKKYSKCDLVTQQCVCLPGYFGNPKEECHGASVFVLVQSSYAVSESI
TQVVIQVKRTGSSSGSVKVTWTATDLTAIHGQDYANSGGILYFNDGDVFKTITIHILNDEKFEA
DEQFAVTLTHVTAGGVLGVLRKATVTIENDDKRCGGKCVFNAHCDEASDKCICNKGLVGDGT
VKCDRKY

>Py_ecY
MAHLLRTIGLFFVINAAILVAPSSGDDYAPVNIPGKGIYVPPKDDYVPPKGGDYAPASGGGYVP
PKGGDYVPATKCCENAVLTNGRCVCKPNYYGSSTHPCVQPCWDKCGINTRCDVNTFKCYCK
DGFIGDPSKGCHVPCHGKCVENASCNPDTDKCECDEGLVGDGTVRCFEPSKVVFAKETYTVS
ESVSTLTVEVLRQGGTIGIVSVTFKATDGTAIHGSDFFNSHGTIVFGEGVSTQYITIYIINDSIYEQ
SETFELTLVSVSVGGEIGEPSTTTITITDNDDACGGKCGPHSHCDGPILLCHCDKGYVKDPYHG
GCTLPCGGRCCTNAYCNTKDNRCYCNEGYYGTPTVNCYQPCKGACKSNAYCGKDNSCCCK
PGFYGDPYVGCTEACNGLCKENARCDIPTQTCQCLPGFYGDPLMGCGKPGQFVFAKSSYSVT
EGSGSVTVTVNRIGSSSGGVAVTWATTDISATHNIDYFNAQGVLFFADGVITRTITFTINADTLY
EVDERFQVTLLSVVPAGGLGSLTITTVTIINDDAACGGPCPVNSYCDKLSQKCICNKGYILYHG
SCVIPCGGKCCANSYCSHKDNQCHCNSGYIGNPTQKCYIPCNNACDDYAYCGKDNKCHCKT
GYYGNPYSKCHLPCNDLCKKYSKCDLVTQQCVCLPGYFGNPKEECHGASVFVLVQSSYAVSE
SVTQVVIQVKRTGSSSGSVKVTWTATDLTAIHGQDYANSGGILYFNDGDVYKTITIHILNDEIFE
ADEQFVVALTHVTAGGVLGVLRKATVTIENDDKRCGGKCVFNAHCDEASNTCICNKGLVGD
GTVKCDLPCGGHCGVYPHSHCNYKTNKCECDDKYHGDPYHGGCICTNGGHTGGSYGSTGG
SHTGGGYGSTGGSHTGGNYGSSSGSYTGGSYGSSSGSHTGGSYGGGNTGGSYGSTGGSHTG
GSYGNTGTGGGYGSTGGSYGGSHTGGSYGNTGGGHSGGHTGGSY

>Py_ec7
MAIRKLSQGYGLWICVLLAATYADHIQDYGGGGGFVDGGAGGGYGGPSHVVVGGYDDHSH
GGGGVVYDDHSNVGGGGYGDASHGGGGVVYDDHSYVGGGGYGGASHGGGGEVFVDHSH
GGGGGYDDHSHGGSGGGKTHCCDNADLINGRCVCKANYRGHSTHPCDKPCWDKCGTNTY
CNVQEFKCYCKEGYIGDPYNECDLPCGGKCASNAYCDSVTNTCRCNTGLIGDATQKCFSPSVI
AFAQSSYTVRESVGVLKIKVTRQGGTFGSITVGWTVSGGTATSPSDFTFALGSLQFGNGDSVEF
ISVKIVNDQAFEPDESFTVTLTSVTSGGTIGSPDVTTVTITNDDKPCGGKCKANSYCRESDNTC
VCNTGYIGDPLRGCKRPCDDKCKKNSYCAPHDNRCYCNKGLVGNPYKEGCDVPCGGKCVL
NSFCRERDNKCYCNEGYVGDGYVRCAKPCDDACRHNAYCNKRDDTCYCNKGYIGDPKVGC
ELPCGGKCASTAYCDSASNTCKCKKGLVGDPHVRCGKPCDNKCRKNAFCNGNNKCQCNPGF
VGDPYEGCDRPCGGKCKTNAYCDEHTNTCKCNKGYIGDPLKKCFEPSVFQFVRRAYTVSESA
GSVDIEVARTSGSEGSYTVSWEANDGSARQPSDYVNGQGTVNFKTNEKTEKITIKIVNDQRNV
VALVELTPIVIPLHKSVSVTSDSSYSVDLVPAHAEDHADQTRDVTVTPTNVFVSKDIGYSTEVV
FYHVEDLVARTNVATVQLTNVSVIQDTSVTKDHVPVST

>Py_ec6
MTYSVSESAGTVSFQLSRTGGLFGSVTVSWSTHDGTARYPSDFSRNSGVATFGNGQSTTTFSIG
VVNDQVYESNESFTVTLSVAVSSTNVVIGTRREATVTITNDDAPCGGKCGPNARCDLTTQTCI

CNQGYRRFHGSTCQLPCGGPCGPNERCDSVTNQCVCIERYFKFRGSCVIPCGGPCGPNAQCD
RVSNQCVCNTGYIRFQRACTLPCGGKCPSNSVCSSSDNRCHCNHGFQGDPYHDGCHLRSVVE
FGQLIYTVSESAGTLSLQLRRTGGLFGSVTVSWSTQNGNANYPSDFSDNSGVATFGNGQSTTT
FTIGIVNDQTYESPEAFKVTLSVSQGSNTNIVVIGERRVATVTIISDDPPCGGPCGLNARCDVHS
QKCVCIEGYTLYDGSCQLPCGGPCGQNAYCNQATNQCVCNTGYFLYHGSCALPCNGACVYN
AYCDKGSNTCKCNHGLVGDPTKKCGIPCDGRCGSFPHSHCDHSTNRCACDTGYAGNPYGSV
GCVCNGGHSGGGGGGDSYVGGSSGGSFHGGSTGGSYAGSSTGDSYTGSSSGGSFHGSSSGGG
YQPAHVGDY

>Py_ecX
PGVVVIASPTYSVVENVGVVVITVTRTGSTYAAVTVKWESRDITALQGSDYIGKYGYIYFASG
QTSETISIEITVDLIYEKDETFSVSLTHVTAGAVLGNIFQTIVTIKNDDPPCGGPCKPFSRCDVPS
QRCVCIKGYIPNSYGGCSLPCGGKCCANAYCDATDNRCKCRTGYIGIGTWKCSKPCDAACKS
YAYCAKDNRCYCSKGYYGSPYKGCHLPCYNLCKANTACDLSNHRCYCKRGYYGNPLKGCS
TPGSFSFTKSVYTVKENVGLVHVTIHRKGSSSQGVSVYWKTVEGSAKRVSDFGDTGGILYFAS
GEIEKTITIHILDDKVYETEESFTVSLTLVSAGGVLGQVVTATITIQDDDDTCGGPCGHYAHCDL
RIHKCVCDKGYLLDSYHGGCVPPCGGRCCNNAYCDRGDNTCKCKPGYLGLGTVACYKPCQ
GACVINAECGPDNKCCCKKGYYGNPYVECTLPCRAQCKRNARCDISSQRCECAHGFYGDPT
VGCDKASTFVFAKPSYQVNEDAGTITVTVNRIGSTYGSISVSWVTKDLSAGHPHDFLNSYGVL
FFAHGVESRTITIYLFVDKVYEQDENFQIVLTKVTPGGVLGGLTATTVTILNDDKPCGGPCGIN
AYCDKPTQKCFHVVENVAPMHTVAPRTTNATVTEATSEPQHRNVTVIPCNRACKDYAFCGKD
NKCHCKTGYYGNPYQTCHLPCHGQCKQNSKCNTTTHQCVCLPGFFGDGKVGCTKPSVFIIAQ
SSYAVKESVNVLVVNVNRIGSSSGSVKVTWKTTDLTAESGHDYVNTGGVLYFGDKETSKDISI
HIIDDKFYEADEQFVITLTEVTQGGVIGLQYKATVTIENDDEPCGGPCGINAFCDKPSQKCLCL
KGFVKWNGIRVVDIARPTHTLTVTTRLTNVSVTTTTQVMPTTEDVYALVEDIPEDMGKLKPVA
IMEVLAEVAVTKLRPTVDIKLHR

>Py_ec12
MFHSIPTLGFCFTLCVLSLFGTTQGTYPIRCCSGSTLINRRCVCNPGYHGDSAHRCVEPCWKK
CKANTICNKNTFKCSCKPGFVGNPYGGCILKATFQFARSSYVVKESAGVVKLQVDRISGGLH
KVIMYWKSTSITARLNSDYRGASGSVTFGVGEKWKFIQIPIVNDQIYERIETFKVTLTSASAGG
AIGTRSVTTVTITDDDLPCGGKCKNYRFSVCNEATNKCVCIKNYYGDPYHGGCRCGGGGGY
